# Evaluation of pharmacological activities and active components in *Tremella aurantialba* by instrumental and virtual analyses

**DOI:** 10.3389/fnut.2022.1083581

**Published:** 2022-12-07

**Authors:** Yonghuan Yan, Mengtian Wang, Xiaoruo Gan, Xu Wang, Chenghao Fu, Yuemin Li, Ning Chen, Pin Lv, Yan Zhang

**Affiliations:** ^1^Hebei Key Laboratory of Forensic Medicine, School of Forensic Medicine, Hebei Medical University, Shijiazhuang, China; ^2^Hebei Food Inspection and Research Institute, Hebei Food Safety Key Laboratory, Key Laboratory of Special Food Supervision Technology for State Market Regulation, Hebei Engineering Research Center for Special Food Safety and Health, Shijiazhuang, China; ^3^Key Laboratory of Neural and Vascular Biology of Ministry of Education, Department of Cell Biology, Cardiovascular Medical Science Center, Hebei Medical University, Shijiazhuang, China

**Keywords:** *Tremella aurantialba*, component analysis, active compounds, drug targets, biological activity, virtual screening

## Abstract

As a kind of medicinal and edible homologous fungus, there is a lack of data on the medicinal value of *Tremella aurantialba*. In this study, ultra-performance liquid chromatography-quadrupole-time of flight-mass spectrometry (UPLC-Q-TOF/MS) was used to screen the chemical components in *T. aurantialba*. Then, network pharmacology was used to reveal the potential biological activities, active compounds, and therapeutic targets of *T. aurantialba*. Finally, the potential binding sites of the active compounds of *T. aurantialba* and key targets were studied by molecular docking. Results showed that 135 chemical components in *T. aurantialba*, especially linoleic acid, and linolenic acid have significant biological activities in neuroprotective, anticancer, immune, hypoglycemic, and cardiovascular aspects. The existence of these bioactive natural products in *T. aurantialba* is consistent with the traditional use of *T. aurantialba*. Moreover, the five diseases have comorbidity molecular mechanisms and therapeutic targets. The molecular docking showed that linolenic acid, adenosine, and vitamin D2 had higher binding energy with RXRA, MAPK1, and JUN, respectively. This study is the first to systematically identify chemical components in *T. aurantialba* and successfully predict its bioactivity, key active compounds, and drug targets, providing a reliable novel strategy for future research on the bioactivity development and utilization of *T. aurantialba*.

## Introduction

*Tremella aurantialba* is a well-known medicinal and edible plant belonging to *fungi*, *Basidiomycota*, *Basidiomycotina*, *Tremellales*, *Naemateliaceae*, *Naematelia Fr* (1). It is widely distributed in Asia and Europe, North and America and Oceania; and can now be found all over the world due to artificial planting. *Tremella aurantialba* is rich in a variety of nutrients such as polysaccharide, dietary fiber, protein and other nutrients, and has great health value. As a high-quality precious medicinal and edible fungi, pharmacological effects of *Tremella aurantialba* have long been mentioned in TCM books. According to the “Compendium of Materia Medica” of the Ming Dynasty, *Tremella aurantialba* traditionally served to treat multiple diseases, especially moistening the lung and relieving cough, protecting the liver and tonifying the kidney. It is mentioned in Famous Doctor Bielu that *Tremella aurantialba* also has the effects of nourishing Qi and prolonging life, invigorating brain and dispersing cold. Xizang Common Chinese Herbal Medicine also records the effects of *Tremella aurantialba* on asthenia tuberculosis cough, hemoptysis, tuberculosis, asthma, hypertension and chronic bronchitis in the elderly. In Asian and European countries, *Tremella aurantialba* has been used as an edible food source and traditional medicine for millenary. The identification of functional components of edible fungi and the exploration of their functions have become the new hot spots and new trend in recent years (2–5). However, there are few modern studies on the compositions and activities of *Tremella aurantialba*, which limit the development and utilization of *Tremella aurantialba*.

Phytochemical profile analysis is a key step in the development and utilization of plant resources and quality safety assurance. So far, research on the composition of *Tremella aurantialba* has mainly involved the nutritional composition (6, 7), polysaccharides (8, 9), volatile components (6, 10) on the fruiting body, mycelium and fermentation broth, while several literatures reported the small-molecule chemical composition of *Tremella aurantialba*. Li isolated and purified 19 monomer compounds from petroleum ether, ethyl acetate and butyl alcohol extracts of the fruiting body of *Tremella aurantialba* by means of atmospheric pressure or vacuum silica gel column chromatography, thin layer chromatography, recrystallization and Sephadex LH-20 gel column chromatography, and finally 13 monomer compounds were identified for the first time through their physical and chemical properties and various spectral data. Among them, 3β-hydroxyl-24α-lanoster-31-*O*-α-D-glucose-8, 24-dien is a new compound (11). However, the above studies adopted the traditional mode of “separation, enrichment, purification and identification” of monomer compounds, ignoring the overall analysis of the chemical profile of *Tremella aurantialba*, which was not conducive to the comprehensive excavation of the active components of *Tremella aurantialba*.

More importantly, the various therapeutic effects of *Tremella aurantialba* mentioned in Chinese medicine books have rarely been confirmed by modern pharmacological studies. Several studies have reported the biological activities of the crude extracts or related products of *Tremella aurantialba in vivo* or *in vitro*. Liu et al. found that the crude lipids extract of *Tremella aurantialba* could promote the penetration of Evans blue through the blood-brain barrier (12). Du et al. found that the chloroform extract of *Tremella aurantialba* fruiting body has a good inhibitory effect on neoplasm cells L210 and SW620 (13). In addition, due to the high content of polysaccharides in *Tremella aurantialba*, more modern studies have emphasized the bioactivities of *Tremella aurantialba* polysaccharide, including enhancing immunity (14), anti-oxidation (9, 15) and so on. However, these studies on the evaluation of the biological activity of *Tremella aurantialba* were not comprehensive, and they only briefly evaluated the role of *Tremella aurantialba* or its main component polysaccharide in some diseases or some of its active functions. Furthermore, the mechanism of *Tremella aurantialba* in the prevention and treatment of diseases is still unclear.

So far, many studies have shown diverse health effects of fungi due to the presence of a variety of bioactive compounds (16–19). These compounds tend to be biologically multiple-functional. However, due to the unclear chemical profile of *Tremella aurantialba*, the biological activities of *Tremella aurantialba* cannot be further excavated at present. Thus, to better apply *Tremella aurantialba* resources, it is urgent to comprehensively analyze the chemical components and biological activities of *Tremella aurantialba*. The main purpose of this study here was to systematically evaluate the chemical composition and bioactivity of *Tremella aurantialba* using UPLC-Q/TOF MS system combined with network pharmacology that have never been systematically investigated. The key active components in *Tremella aurantialba* and their pharmacodynamic targets were also revealed. Whether the component binds to the target were validated by molecular docking. Moreover, the comorbidity mechanisms and potential therapeutic targets of five diseases were explored. In this direction, the research aims to provide some knowledge on the chemical composition and bioactivities of *Tremella aurantialba* from the health point of view, which will help to verify its clinical application and the further development of *Tremella aurantialba* resources.

## Materials and methods

### Chemicals and reagents

Liquid chromatography-mass spectrometry (LC-MS) grade methanol, acetonitrile, ammonium formate and formic acid were purchased from Sigma-Aldrich (St. Louis, MO, USA). Deionized water (18.3 MΩ) was generated by a Milli-Q water purification system (Millipore Ltd., Bedford, MA, USA). *Tremella aurantialba* were collected from Yunnan Bacteria Horizon Biotechnology Co., Ltd.

### Samples preparation

*Tremella aurantialba* samples was frozen dried under vacuum condition and crushed into powder through a 100-mesh screen. Then the samples were kept at –80°C for further use. The extraction procedure and conditions were performed as following: 1.0 g of freeze drying *Tremella aurantialba* powder was weighted and placed in 50 mL centrifuge tube, and then the sample was extracted with 20 mL methanol-water (7: 3, V/V) (Darmstadt, Germany). After vortexing for 1 min and sonicating at room temperature for 30 min, the mixture was centrifuged at 10 000 r/min at 4°C for 5 min. 1 mL supernatant was filtered with a 0.22 μm nylon membrane before UPLC-Q-TOF/MS analysis.

### Instrumentation

A LC-30AD UPLC system (Shimadzu Corporation, Kyoto, Japan) was used for the chromatographic separation of the samples. Compounds separation was performed on a Waters ACQUITY UPLC HSS T3 column (100 mm × 2.1 mm, 1.8 μm) using a gradient elution consist of 0.01% formic acid + 2 mmol/L ammonium formate (A) and acetonitrile + 0.01% formic acid + 2 mmol/L ammonium formate (B). The gradient program was: 5–12% B at 0–5 min, 12–55% B at 5–7 min, 55–65% B at 7–10 min, 65–98% B at 10–20 min, 98% B at 20–25 min, 98–5% B at 25–25.1 min and 5% B at 25.1–28 min. The flow rate was 0.3 mL/min, the column temperature was maintained at 40°C. The sample injection volume was 2 μL.

The QTOF MS analysis, controlled by the Sciex OS software (version 1.5.0, Sciex, USA), was performed on a hybrid quadrupole time-of-flight tandem mass spectrometer Q-TOF/MS with an electrospray ionization (ESI) source (Triple TOF™ 5600 + MS system, AB Sciex Corporation., Foster City, CA, USA). The ionization of compounds was in the positive or negative mode. Information dependent acquisition method was used for acquiring spectra data with a scan range from 100 to 1000 *m/z*. Other optimized MS parameters were set as follows: ion spray voltage 5500 V in positive ion mode and 4500 V in negative ion mode; the ion source gas1, 50 psi; the ion source gas 250 psi; the curtain gas, 35 psi; ion source temperature, 500°C; declustering potential, 80 V; collision energy, 60 V.

### Network pharmacology analysis

#### Target prediction of *Tremella aurantialba*

First, the active components of *Tremella aurantialba* were screened by SwissADME platform^1^ and literatures. At SwissADME platform, gastrointestinal absorption, one of the pharmacokinetic parameters, was set as “HIGH” as the condition for drug absorption and active compounds with good oral bioavailability were screened. At SwissADME platform, the screening criteria for bioavailability are lipophilicity: XLOGP3 between –0.7 and +5.0, size: MW between 150 and 500 g/mol, polarity: TPSA between 20 and 130 Å^2^, solubility: log S not higher than 6, saturation: fraction of carbons in the sp3 hybridization not less than 0.25, and flexibility: no more than 9 rotatable bonds. Besides, the drug-likeness is also considered. For cosmeceutical parameters (Lipinski, Ghose, Veber, Egan, Muegge), two or more of them with “YES” can be regarded as active components. At the same, the components with significant pharmacological activity reported in the literature are also considered as active ingredients. Secondly, Swiss Target Prediction platform^2^ was applied to predict the possible targets. Swiss Target Prediction selected the targets whose probability is >0.12 in the prediction results for further analysis. At the same time, experimentally verified targets information was downloaded from NPASS^3^ and the entries related to the active components of *Tremella aurantialba* were extracted. Finally, target information was integrated and accumulated to obtain the possible targets of *Tremella aurantialba* active components.

#### Prediction for targets of five diseases

Data for all-associated disease targets were acquired from six databases, including the National Center for Biotechnology Information database (NCBI^4^), the Online Mendelian Inheritance in Man Database (OMIM^5^), the GeneCards database,^6^ the Therapeutic Target Database (TTD^7^), the Comparative Toxicogenomics Database (CTD^8^), a database of gene-disease associations (DisGNet^9^), using “Nervous system disease,” “Immune System Diseases,” “Endocrine System Diseases,” “Neoplasms,” “Cardiovascular Diseases” and their synonyms and descendants in the CTD Database as the keywords, respectively. The above targets were converted and queried into the UniProt ID format with “Homo sapiens” as the qualifying condition in the UniProt database.^10^ Finally, the gene library of all was established by eliminating repeated targets.

#### Intersection between active compounds and disease targets

The intersection targets between the disease genes and the predicted *Tremella aurantialba* targets were obtained and the National Genomics Data Center website^11^ was used to construct a Venn diagram for visualization.

#### Protein-protein interaction network construction

The intersection targets above were imported into the STRING database^12^ for protein interaction network analysis. The screening condition of the species was set to “Homo sapiens” and the minimum required interaction score was “highest confidence (0.9)”. Input protein-protein interaction (PPI) information into Cytoscape 3.7.1^13^ for visualization and constructs network of potential key targets.

#### Topological and cluster analyses of the protein-protein interaction network

The CytoHubba plugin in Cytoscape was used to identify hub genes. Three critical topological parameters were chosen for screening the core composite targets based on the PPI network: degree (D), betweenness (B) and closeness (C). Values for the three parameters indicated the significance as well as the impact of relevant nodes in the entire network. The top 10 nodes were set as the core targets and the hub gene was obtained through the intersection of the top 10 core targets obtained by different algorithms. The MCODE plug-in in Cytoscape was used to screen PPI network modules using various cut-offs: degree = 2, k-core = 2, node score = 0.2, and max depth = 100.

#### Gene ontology and kyoto encyclopedia of genes and genomes enrichment analyses

The intersection targets above were subjected to the Gene Ontology (GO) biological process analysis and the Kyoto Encyclopedia of Genes and Genomes (KEGG) enrichment analyses using the DAVID database,^14^ with FDR < 0.05 and *P* < 0.05 as cut-off values. R version 3.4.1 was used to visualize the results.

#### Construction of active compound-target network

After obtaining the intersection genes, reverse screening the active components in *Tremella aurantialba*. For visualization, potential active components and matching intersection targets were imported into Cytoscape 3.7.1 software and a network of compound-target network was built. Each component of targets is represented by nodes and the relationship between the components, diseases and the targets are represented by connecting lines.

### Verification of the compound-target interactions

The crystal structures of the targets and the chemical structures of the composition were obtained from the PDB^15^ and the PubChem.^16^ Molecular docking was performed using the AutoDock software. The water molecules and atoms were removed from the target receptors and then the affinities were obtained. Finally, the binding sites of composition and targets were visualized by the PyMOL software.

## Results and discussion

### Targeted and untargeted analysis of chemical components in *Tremella aurantialba*

In this study, targeted and untargeted analysis strategies combined with UPLC-Q/TOF MS were firstly used for the qualitative screening of chemical components in *Tremella aurantialba* and the chromatography condition was optimized to obtained higher peak capacity, shorter retention and better resolution of components in *Tremella aurantialba*. Subsequently, a total of 135 chemical components were rapidly identified by comparing with TCM MS/MS database, online Chemspider database or inferred through mass spectrometry fragment ion analysis and literature data, including 22 organic acids, 20 amino acids and their derivatives, 12 fatty acids, 13 saccharides, 8 nucleosides, 6 vitamins, 7 alkaloids, 8 esters, 4 amides, 3 terpenoids, 2 phenols, 2 ethers, 1 alcohol, 1 ketone and 26 other classes (Table 1). Among these components, fatty acids, organic acids and saccharides were the major components of *Tremella aurantialba*. In addition, the species of amino acids in *Tremella aurantialba* are abundant. The total ion chromatograms of *Tremella aurantialba* are shown in Supplementary Figure 1. By comparing with the available credible standards and literatures, and cross-checking with some available spectral databases, TCM, Metlin and Chemspider, the components of *Tremella aurantialba* were identified and characterized. It could be summarized as follows and some specific compounds were taken as examples.

**TABLE 1:** Identification of components in *Tremella aurantialba* by UPLC-Q/TOF MS.

No.	Compounds	Formulas	Adducts	Precursor ions *m/z*	Peak area	Retention time/min.	Compound types
1	Linoleic acid	C_18_H_32_O_2_	[M-H] ^–^	279.2341	6.00E + 07	19.92	Fatty acids
2	Oleic acid	C_18_H_34_O_2_	[M-H] ^–^	281.2496	4.31E + 07	20.64	Fatty acids
3	Palmitic acid	C_16_H_32_O_2_	[M-H] ^–^	255.2335	9.15E + 06	20.35	Fatty acids
4	Hydroperoxy-octadecadienoic acid isomer 1	C_18_H_32_O_4_	[M-H] ^–^	311.2236	7.21E + 06	15.14	Fatty acids
5	Linolenic acid	C_18_H_30_O_2_	[M + H] ^+^	279.2313	2.89E + 06	16.79	Fatty acids
6	Stearic acid	C_18_H_36_O_2_	[M-H] ^–^	283.2648	1.75E + 06	21.49	Fatty acids
7	Palmitoleic acid	C_16_H_30_O_2_	[M-H] ^–^	253.2175	8.67E + 05	19.45	Fatty acids
8	Trihydroxyoctadecenoic acid	C_18_H_34_O_5_	[M-H] ^–^	329.2331	2.05E + 05	11.99	Fatty acids
9	Lignoceric acid	C_24_H_48_O_2_	[M-H] ^–^	367.3576	9.00E + 04	25.42	Fatty acids
10	Arachidic acid	C_20_H_40_O_2_	[M-H] ^–^	311.2956	7.39E + 04	22.58	Fatty acids
11	Behenic acid	C_22_H_44_O_2_	[M-H] ^–^	339.3267	7.38E + 04	23.84	Fatty acids
12	Hydroxystearic acid	C_18_H_35_O_3_	[M-H] ^–^	298.2501	6.97E + 03	8.58	Fatty acids
13	Citric acid	C_6_H_8_O_7_	[M-H] ^–^	191.0197	4.83E + 06	0.98	Organic acids
14	Maleic acid	C_4_H_6_O_5_	[M-H] ^–^	133.0145	1.24E + 06	0.91	Organic acids
15	Galactonic acid	C_6_H_12_O_7_	[M + H] ^+^	197.0653	9.67E + 05	0.84	Organic acids
16	Phthalic acid	C_8_H_6_O_4_	[M + H] ^+^	167.0334	8.55E + 05	21.16	Organic acids
17	Indoleacrylic acid	C_11_H_9_NO_2_	[M + H] ^+^	188.0707	6.78E + 05	5.42	Organic acids
18	Amber Acid	C_4_H_6_O_4_	[M-H] ^–^	117.0194	1.38E + 05	1.5	Organic acids
19	Hydroxymethylglutaric acid	C_6_H_10_O_5_	[M-H] ^–^	161.0455	5.18E + 04	0.9	Organic acids
20	Ursolic acid	C_30_H_48_O_3_	[M-H] ^–^	455.3527	5.10E + 04	22.47	Organic acids
21	3-Phenylbutyric acid	C_10_H_12_O_2_	[M + H] ^+^	165.0905	4.02E + 04	7.56	Organic acids
22	Oleanolic acid	C_30_H_48_O_3_	[M-H] ^–^	455.3515	3.57E + 04	22.45	Organic acids
23	D-Glucose 6-phosphate	C_6_H_13_O_9_P	[M + H] ^+^	261.0363	3.40E + 04	0.85	Organic acids
24	Sinapic acid 4-*O*-glucoside	C_17_H_22_O_10_	[M-H] ^–^	385.1149	3.24E + 04	0.87	Organic acids
25	Phenyllactic acid	C_9_H_10_O_3_	[M-H] ^–^	165.0558	2.08E + 04	8.17	Organic acids
26	2-Aminoisobutyric acid	C_4_H_9_NO_2_	[M + H] ^+^	104.0704	1.82E + 04	0.84	Organic acids
27	Aconitic acid	C_6_H_6_O_6_	[M-H] ^–^	173.0094	1.09E + 04	0.98	Organic acids
28	Quinic acid	C_7_H_12_O_6_	[M-H] ^–^	191.056	9.64E + 03	0.94	Organic acids
29	4-Hydroxybenzoic acid	C_7_H_6_O_3_	[M + H] ^+^	139.0387	8.59E + 03	0.95	Organic acids
30	3-*O*-caffeoyl-shikimic acid	C_16_H_16_O_8_	[M-H] ^–^	335.0783	8.59E + 03	1.84	Organic acids
31	cinnamic acid	C_9_H_8_O_2_	[M + H] ^+^	149.0595	4.55E + 03	13.41	Organic acids
32	*p*-Anisic acid	C_8_H_8_O_3_	[M + H] ^+^	153.0546	4.00E + 03	9.56	Organic acids
33	2,4-dihydroxybenzoic acid	C_7_H_6_O_4_	[M-H] ^–^	153.0198	1.15E + 03	1.02	Organic acids
34	Shikimic acid	C_7_H_10_O_5_	[M-H] ^–^	173.0455	1.07E + 03	0.94	Organic acids
35	L-Carnitine	C_7_H_15_NO_3_	[M + H] ^+^	162.1125	3.69E + 06	0.82	Amino acids
36	L- (+)-Valinol	C_5_H_13_NO	[M + H] ^+^	104.1072	1.91E + 06	0.82	Amino acids
37	L-Leucine	C_6_H_13_NO_2_	[M + H] ^+^	132.1018	1.27E + 06	1.7	Amino acids
38	L-aspartic acid	C_4_H_7_NO_4_	[M + H] ^+^	134.0448	1.34E + 05	0.85	Amino acids
39	L-Methionine	C_5_H_11_NO_2_S	[M + H] ^+^	150.0578	7.39E + 04	1.18	Amino acids
40	Pipecolic acid	C_6_H_11_NO_2_	[M + H] ^+^	130.0862	3.88E + 04	0.93	Amino acids
41	L-tyrosine	C_9_H_11_NO_3_	[M + H] ^+^	182.0812	2.90E + 04	0.95	Amino acids
42	Histidine	C_6_H_9_N_3_O_2_	[M + H] ^+^	156.0765	2.58E + 04	0.94	Amino acids
43	L-Alanine	C_3_H_7_NO_2_	[M + H] ^+^	90.0547	2.03E + 04	5	Amino acids
44	Isoleucine	C_6_H_13_NO_2_	[M + H] ^+^	132.1018	2.02E + 04	1.77	Amino acids
45	L-Aspartyl-L-phenylalanine	C_13_H_16_N_2_O_5_	[M + H] ^+^	281.113	1.68E + 04	2.39	Amino acids
46	Phenylalanine	C_9_H_11_NO_2_	[M + H] ^+^	166.0861	1.67E + 04	0.97	Amino acids
47	Arginine	C_6_H_14_N_4_O_2_	[M + H] ^+^	175.119	1.59E + 04	0.94	Amino acids
48	L-Glutamic acid	C_5_H_9_NO_4_	[M + H] ^+^	148.0604	1.55E + 04	0.93	Amino acids
49	Glutamine	C_5_H_10_N_2_O_3_	[M + H] ^+^	145.0617	1.40E + 04	0.88	Amino acids
50	Proline	C_5_H_9_NO_2_	[M + H] ^+^	116.0706	1.15E + 04	0.94	Amino acids
51	Levodopa	C_9_H_11_NO_4_	[M + H] ^+^	198.076	8.80E + 03	0.96	Amino acids
52	Threonine	C_4_H_9_NO_3_	[M + H] ^+^	120.0654	5.92E + 03	0.91	Amino acids
53	Valine	C_5_H_11_NO_2_	[M + H] ^+^	118.0867	5.38E + 03	0.99	Amino acids
54	GABA	C_4_H_9_NO_2_	[M + H] ^+^	104.0703	4.33E + 03	0.87	Amino acids
55	Gluconic acid	C_6_H_12_O_7_	[M-H] ^–^	195.0522	2.77E + 07	0.8	Saccharides
56	Mannitol	C_6_H_14_O_6_	[M-H] ^–^	181.0719	4.40E + 06	0.82	Saccharides
57	D-Sorbitol	C_6_H_14_O_6_	[M-H] ^–^	181.072	3.41E + 06	0.82	Saccharides
58	Sucrose	C_12_H_22_O_11_	[M-H] ^–^	341.1091	2.85E + 06	0.85	Saccharides
59	Trehalose	C_12_H_22_O_11_	[M-H] ^–^	341.1091	2.85E + 06	0.85	Saccharides
60	Xylitol	C_5_H_12_O_5_	[M-H] ^–^	151.0611	5.66E + 05	0.86	Saccharides
61	D- (+) – Mannose	C_6_H_12_O_6_	[M-H] ^–^	179.056	1.26E + 05	0.91	Saccharides
62	Gluconic acid	C_6_H_10_O_7_	[M-H] ^–^	193.0355	7.87E + 04	0.74	Saccharides
63	Melezitose	C_18_H_32_O_16_	[M-H] ^–^	503.1613	5.18E + 04	0.93	Saccharides
64	Beta-N-Acetylglucosamine	C_8_H_15_NO_6_	[M + H] ^+^	222.0973	1.48E + 04	0.88	Saccharides
65	D-xylose	C_5_H_10_O_5_	[M-H] ^–^	149.0459	1.12E + 04	0.9	Saccharides
66	Neoeriocitrin	C_27_H_32_O_15_	[M-H] ^–^	595.1696	4.05E + 03	20.39	Saccharides
67	D- (+) – digitoxose	C_6_H_12_O_4_	[M-H] ^–^	147.0665	2.50E + 03	2.38	Saccharides
68	Adenine	C_5_H_5_N_5_	[M + H] ^+^	136.062	4.75E + 06	1.82	Nucleosides
69	Uridine	C_9_H_12_N_2_O_6_	[M-H] ^–^	243.0622	6.15E + 05	1.83	Nucleosides
70	Uracil	C_4_H_4_N_2_O_2_	[M + H] ^+^	113.0342	3.24E + 05	1.73	Nucleosides
71	Cytidine	C_9_H_13_N_3_O_5_	[M-H] ^–^	242.0791	2.89E + 05	0.79	Nucleosides
72	Hydroxypurine	C_5_H_4_N_4_O	[M + H] ^+^	137.0456	1.10E + 05	1.55	Nucleosides
73	Adenosine	C_10_H_13_N_5_O_4_	[M + H] ^+^	268.1039	5.45E + 04	4.88	Nucleosides
74	Xanthine	C_5_H_4_N_4_O_2_	[M + H] ^+^	153.0408	5.36E + 04	1.29	Nucleosides
75	Cytosine	C_4_H_5_N_3_O	[M + H] ^+^	112.0502	6.89E + 03	0.95	Nucleosides
76	Nicotinic acid	C_6_H_5_NO_2_	[M + H] ^+^	124.0393	2.57E + 06	0.91	Vitamins
77	Nicotinamide	C_6_H_6_N_2_O	[M + H] ^+^	123.0554	5.42E + 05	2.66	Vitamins
78	Vitamin D2	C_28_H_44_O	[M + H] ^+^	397.3456	2.67E + 05	24.12	Vitamins
79	Pantothenic acid	C_9_H_17_NO_5_	[M-H] ^–^	218.1036	1.92E + 05	4.93	Vitamins
80	γ-Tocotrienol	C_28_H_42_O_2_	[M-H] ^–^	409.3106	1.25E + 05	20.24	Vitamins
81	Vitamin C	C_6_H_8_O_6_	[M-H] ^–^	175.025	9.58E + 03	0.9	Vitamins
82	Trigonelline	C_7_H_7_NO_2_	[M + H] ^+^	138.0551	1.96E + 06	0.86	Alkaloids
83	Choline	C_5_H_13_NO	[M + H] ^+^	104.1072	1.85E + 06	0.82	Alkaloids
84	Nicotine	C_10_H_14_N_2_	[M + H] ^+^	163.1229	9.04E + 04	11.76	Alkaloids
85	(*R*, *S*)-anatabine	C_10_H_12_N_2_	[M + H] ^+^	161.1073	8.14E + 04	14.94	Alkaloids
86	Lupinine	C_10_H_19_NO	[M + H] ^+^	170.1536	1.16E + 04	19.59	Alkaloids
87	Arecoline	C_8_H_13_NO_2_	[M + H] ^+^	156.1017	3.65E + 03	0.96	Alkaloids
88	Stachydrine	C_7_H_13_NO_2_	[M + H] ^+^	144.1012	2.29E + 03	0.95	Alkaloids
89	Methyl linoleate	C_19_H_34_O_2_	[M + H] ^+^	295.2629	1.24E + 06	21.29	Esters
90	C20 sphinganine	C_20_H_43_NO_2_	[M + H] ^+^	330.3358	1.06E + 06	10.76	Esters
91	Phytosphingosine	C_18_H_39_NO_3_	[M + H] ^+^	318.2992	8.80E + 05	9.23	Esters
92	Gluconolactone	C_6_H_10_O_6_	[M + H] ^+^	179.0548	8.17E + 05	1.01	Esters
93	3-N-butyl-4, 5-dihydrophthalide	C_12_H_16_O_2_	[M + H] ^+^	193.1219	8.80E + 04	9.94	Esters
94	Glycerophosphoric acid	C_3_H_9_O_6_P	[M-H] ^–^	171.0065	1.73E + 04	0.77	Esters
95	2-linoleoyl-sn-glycero-3-phosphoethanolamine	C_23_H_44_NO_7_P	[M-H] ^–^	476.2778	1.16E + 04	17.93	Esters
96	1-oleoyl phosphatidylethanolamine	C_23_H_46_NO_7_P	[M-H] ^–^	478.2939	7.86E + 03	18.76	Esters
97	Thraustochytroside-2	C_43_H_79_NO_8_	[M + H] ^+^	738.5864	7.96E + 06	24.47	Amides
98	Thraustochytroside-1	C_42_H_77_NO_8_	[M + H] ^+^	724.5706	3.29E + 06	23.2	Amides
99	Thraustochytroside A	C_41_H_75_NO_8_	[M + H] ^+^	710.5548	1.18E + 06	22.24	Amides
100	Feruloylagmatine	C_15_H_22_N_4_O_3_	[M-H] ^–^	305.1606	1.70E + 05	8.21	Amides
101	Glochidone	C_30_H_46_O	[M + H] ^+^	423.3606	8.95E + 04	11.98	Terpenoids
102	F2 Ginsenoside	C_42_H_72_O_13_	[M-H] ^–^	783.4919	5.36E + 03	17.99	Terpenoids
103	Heliangin	C_20_H_25_O_6_	[M-H] ^–^	360.1563	7.72E + 02	8.18	Terpenoids
104	Dihydrocapsiate	C_18_H_28_O_4_	[M-H] ^–^	307.1909	7.52E + 03	22.72	Phenols
105	Isoacteoside	C_29_H_36_O_15_	[M + NH_4_] ^+^	642.2412	2.63E + 02	21.17	Phenols
106	(+)-Costunolide	C_15_H_20_O_2_	[M + H] ^+^	233.1538	9.34E + 04	7.33	Ethers
107	Di-2-propenyl disulfide	C_6_H_10_S_2_	[M + FA-H] ^–^	191.02	8.95E + 04	0.98	Ethers
108	N-(4-Acetylphenyl) maleimide	C_12_H_9_NO_3_	[M-H] ^–^	214.0513	9.27E + 05	0.8	Ketone
109	Mycosporine serinol	C_11_H_19_NO_6_	[M + H] ^+^	262.1276	2.21E + 05	0.91	Alcohols
110	Glycerophosphocholine	C_8_H_20_NO_6_P	[M + H] ^+^	258.1112	1.86E + 07	0.81	Other classes
111	N1-{[2-(Diethylamino)-1, 3-thiazol-5-yl] methyl}-N2, N2, 4-trimethyl-1, 2-pentanediamine	C_16_H_32_N_4_S	[M-H] ^–^	311.2269	2.27E + 06	10.03	Other classes
112	Metilox	C_18_H_28_O_3_	[M + H] ^+^	293.2107	2.19E + 06	8.45	Other classes
113	2-Dodecyl-N-(1, 2, 2, 6, 6-Pentamethylpiperidin-4-yl) succinimide	C_26_H_48_N_2_O_2_	[M + H] ^+^	421.3803	1.82E + 06	17.28	Other classes
114	N-[2-({[4-(Diethylamino)butyl] carbamothioyl} amino) ethyl] pentanamide	C_16_H_34_N_4_OS	[M-H] ^–^	329.2376	1.57E + 06	8.24	Other classes
115	Phenylacetylene	C_8_H_6_	[M + H] ^+^	103.0545	1.45E + 06	2.53	Other classes
116	1-(Dicyclohexylphosphino)-4-methylpiperazine	C_17_H_33_N_2_P	[M-H] ^–^	295.2321	1.02E + 06	11.63	Other classes
117	Dimethyl (1-{(2E)-3-[4-hydroxy-3,5-bis(2-methyl-2-propanyl)-5,6,7,8-tetrahydro-1-naphthalenyl]-2-propenoyl} cyclopentyl) phosphonate	C_28_H_43_O_5_P	[M-H] ^–^	489.2779	9.71E + 05	7.7	Other classes
118	(2E)-N-Cycloheptyl-3-(4-propoxyphenyl) acrylamide	C_19_H_27_NO_2_	[M-H] ^–^	300.197	6.28E + 05	7.27	Other classes
119	(E)-2-[4-(Dimethylamino)benzyl]-N-hexadecyldiazenecarbothioamide	C_26_H_46_N_4_S	[M-H] ^–^	445.3378	5.38E + 05	17.56	Other classes
120	HexCer 37:2;3	C_43_H_81_NO_9_	[M-H] ^–^	754.5853	5.02E + 05	23.68	Other classes
121	HexCer 36:2;3	C_42_H_79_NO_9_	[M-H] ^–^	740.569	3.65E + 05	23.29	Other classes
122	Ile-Gly-Ile	C_14_H_27_N_3_O_4_	[M + H] ^+^	302.2066	1.33E + 05	7.26	Other classes
123	Leu-Val	C_11_H_22_N_2_O_3_	[M + H] ^+^	231.1699	1.30E + 05	4.71	Other classes
124	Thr-Leu	C_10_H_20_N_2_O_4_	[M + H] ^+^	233.1517	8.34E + 04	7.34	Other classes
125	Thr-Val-Leu	C_15_H_29_N_3_O_5_	[M + H] ^+^	332.217	8.21E + 04	7.26	Other classes
126	Colneleic acid isomer 1	C_18_H_30_O_3_	[M-H]-	293.2123	7.90E + 04	14.31	Other classes
127	Ile-Glu	C_11_H_20_N_2_O_5_	[M + H] ^+^	261.1442	7.11E + 04	1.78	Other classes
128	AC1L1 × 1Z	C_23_H_46_N_6_O_13_	[M + H] ^+^	637.3017	6.10E + 04	17.08	Other classes
129	NCGC00380283-01!4-[5-[[4-[5-[acetyl(hydroxy) amino] pentylamino]-4-oxobutanoyl]-hydroxyamino] pentylamino]-4-oxobutanoic acid	C_20_H_36_N_4_O_8_	[M + H] ^+^	478.2894	5.46E + 04	10.38	Other classes
130	Massbank: RP016503 Ala-Phe| (2*S*)-2-[[(2*S*)-2-azaniumylpropanoyl] amino]-3-phenylpropanoate	C_12_H_16_N_2_O_3_	[M + H] ^+^	237.1224	2.54E + 04	3.12	Other classes
131	11-Hydroperoxy-octadecatrienoic acid	C_18_H_30_O_4_	[M-H] ^–^	309.2069	2.43E + 04	10.36	Other classes
132	Val-Gly-Val	C_12_H_23_N_3_O_4_	[M + H] ^+^	274.1752	1.20E + 04	4.28	Other classes
133	Met-Phe	C_14_H_20_N_2_O_3_S	[M + H] ^+^	297.126	1.04E + 04	6.7	Other classes
134	Lauryl hydrogen sulfate	C_12_H_26_O_4_S	[M-H] ^–^	265.1475	3.26E + 03	15.03	Other classes
135	Fraxin	C_16_H_18_O_10_	[M-H] ^–^	369.0841	2.11E + 03	0.86	Other classes

#### Identification of fatty acids

**FIGURE 1:**
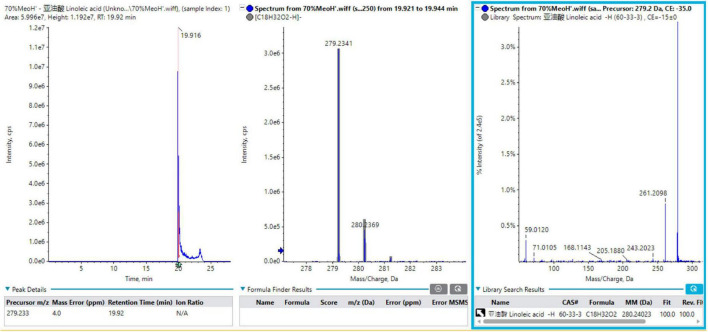
The mass spectra, fragment information and Sciex OS screening interface of linoleic acid in negative mode.

A total of 12 fatty acids mainly originated from *Tremella aurantialba*, including linoleic acid, oleic acid, palmitic acid, hydroperoxy-octadecadienoic acid isomer 1, linolenic acid, stearic acid, palmitoleic acid, trihydroxyoctadecenoic acid, lignoceric acid, arachidic acid, behenic acid and hydroxystearic acid. In order to better understand the MS fragmentation pattern of fatty acids from *Tremella aurantialba* constituents, we took compound 1 as an example, which showed [M-H]^–^ ion at *m/z* 279.2341 on the TOF-MS spectrum. The molecular formula was speculated to be C_18_H_32_O_2_ based on the analysis of its elemental composition and fractional isotope abundance. The main fragment ions were observed at *m/z* 261.2098 [M-H-H_2_O]^–^, 243.2023 [M-H-2H_2_O]^–^, 205.1880 [M-H-C_3_H_6_O_2_]^–^ in the negative ion spectrum by the TOF-MS/MS screening. These fragments were coincided with the linoleic acid in the TCM MS/MS database and the reference substance linoleic acid. As such, compound 1 was finally identified to be linoleic acid. The mass spectra, fragment information and Sciex OS screening interface of linoleic acid in negative mode were illustrated in Figure 1.

For the components not in the TCM MS/MS database, analysis was conducted using the XCMS online (Metlin) database, fragmentation pathway and literature data. We took compound 3 as an example, which showed a protonated ion [M-H]^–^ at *m/z* 255.2335 with the molecular formula C_16_H_32_O_2_ in the negative ionization mode. The hydrogen adducts [M-H] ^–^ were observed in negative ionization mode. Further MS/MS scan showed that they produced fragment ions at *m/z* 237.2087, 201.8310, etc. Compound 3 was identified as palmitic acid after comparison with available MS data in the literature (20). The mass spectra and fragment information and Sciex OS screening interface were illustrated in Supplementary Figure 2.

#### Identification of organic acids

A total of 22 organic acids mainly originated from *Tremella aurantialba*. We took compound 13 as an example. Based on the analysis of its elemental composition and fractional isotope abundance, its molecular formula was predicted to be C_6_H_8_O_7_. The precise molecular weight was 192.0270, and the main fragment ions were analyzed via the MS/MS screening and observed at *m/z* 129.0115 [M-H-H_2_O-CO_2_]^–^, 111.0018 [M-H-2H_2_O-CO_2_]^–^, 85.0241 [M-H-H_2_O-2CO_2_]^–^ and 67.0143 [M-H-2H_2_O-2CO_2_]^–^ in the negative ion spectrum. It was identified as citric acid by searching the database, inferred through mass spectrometry fragment ion analysis and literature data (21). The mass spectra, fragmentation pathway and Sciex OS screening interface of citric acid in negative mode are illustrated in Supplementary Figure 3.

#### Identification of saccharides

A total of 13 saccharides mainly originated from *Tremella aurantialba*. Took compound 56 as an example, the precise molecular weight was 181.0790, and the molecular formula was speculated to be C_6_H_14_O_6_ based on the analysis of its elemental composition and fractional isotope abundance. The main fragment ions were analyzed via the MS/MS screening and observed at *m/z* 163.0508 [M-H-H_2_O]^–^, 119.0260 [M-H-C_2_H_6_O_2_]^–^, 101.0172 [M-H-2H_2_O-CO_2_]^–^, 89.0186 [M-H-C_3_H_8_O_3_]^–^, 71.0092 [M-H-C_3_H_8_O_3_-H_2_O]^–^, 59.0101 [M-H-C_4_H_10_O_4_]^–^. Compound 56 was identified as mannitol by comparing with TCM MS/MS database and literature data (22). The mass spectra, fragment information and Sciex OS screening interface were illustrated in Supplementary Figure 4.

#### Identification of others

In addition, other compounds have been identified from *Tremella aurantialba*, including amino acids, nucleosides and vitamins, etc. Compound 76 was selected as an example. The precise molecular weight was 124.0393, and the main fragment ions were analyzed via the MS/MS screening and observed at *m/z* 106.0280 [M + H-H_2_O]^+^, 80.0498 [M + H-CO_2_]^+^, 53.0413 [M + H-C_3_H_3_O_2_]^+^ in the positive ion spectrum (23). Based on the analysis of its elemental composition and fractional isotope abundance, its molecular formula was predicted to be C_6_H_5_NO_2_. According to the fragment information and TCM MS/MS database, the compound was identified as nicotinic acid. The secondary fragment of this substance was consistent with the secondary fragment of the reference substance in the TCM MS/MS database. The mass spectra, fragment information and Sciex OS screening interface of nicotinic acid in positive mode are illustrated in Supplementary Figure 5.

### Biological activities analysis of *Tremella aurantialba*

**FIGURE 2:**
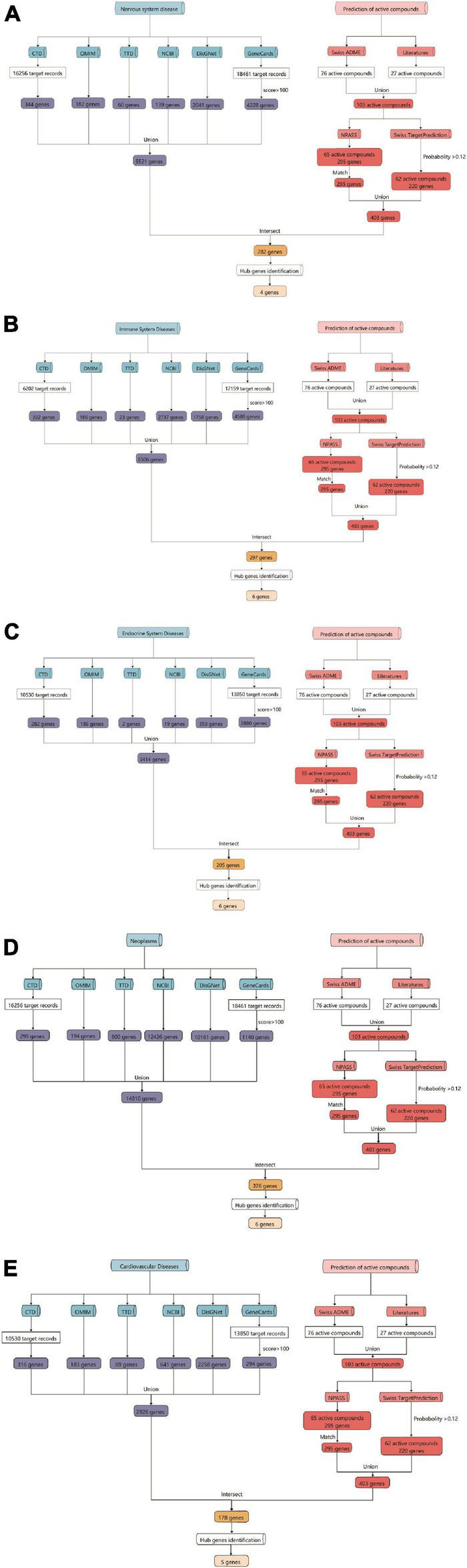
Flow chart of the prediction for targets of active components from *Tremella aurantialba* by network pharmacological analysis. **(A)** *Tremella aurantialba-*nervous system diseases; **(B)** *Tremella aurantialba-*immune system diseases; **(C)** *Tremella aurantialba-*endocrine system diseases; **(D)** *Tremella aurantialba-* neoplasm system diseases; **(E)** *Tremella aurantialba-*cardiovascular system diseases.

**FIGURE 3:**
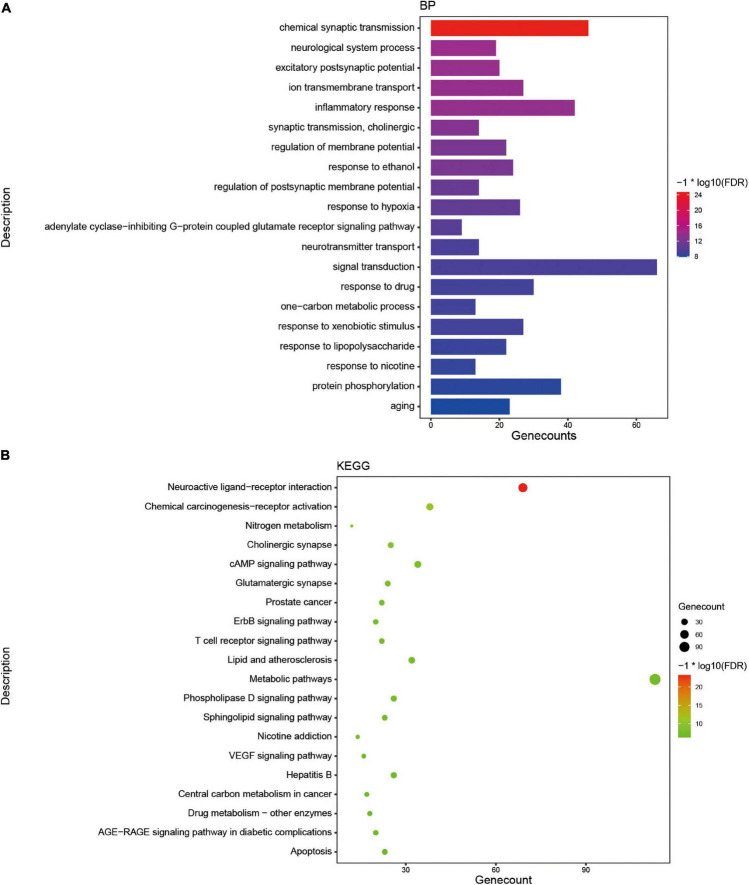
The enrichment analysis of targets of active components from *Tremella aurantialba*. **(A)** Histogram of GO enrichment analysis of targets. **(B)** Bubble chart of KEGG enrichment analysis of targets.

As a functional food raw material or traditional Chinese medicine formula, *Tremella aurantialba* is put into use as a whole. There are many kinds of compounds with complex systems, and the bioactivity evaluation and mechanism study of single component cannot truly reflect the overall physiological effects of *Tremella aurantialba*. Here, the potential bioactivity of *Tremella aurantialba* was also systematically predicted by enrichment analysis of the targets of active components. As shown in Figure 2 and Supplementary Table 1, Swiss ADME database was used to screen the gastrointestinal absorbance and drug-like properties of the identified chemical components of *Tremella aurantialba*, and 76 active components were obtained. In addition, this study also conducted literature research on the pharmacological activities of the identified components in *Tremella aurantialba*. The results are shown in Supplementary Table 2. The existence of these bioactive natural products in *Tremella aurantialba* is consistent with the traditional use of *Tremella aurantialba*. Then, 27 components that do not meet the ADME screening criteria but have clear pharmacological effects are also included in the list of active components, in order to more comprehensively identify the efficacy and material basis of *Tremella aurantialba*. Swiss TargetPrediction database was used to predict the targets of 101 active components, and 62 active compounds and their corresponding gene targets were obtained. At the same time, the active components were further verified by NPASS database, and 65 active compounds and 295 corresponding protein targets were screened, and 295 gene targets were obtained after the protein targets were matched. Finally, 403 putative targets related to the active components of *Tremella aurantialba* were obtained from the two databases. In order to elucidate the biological activity of *Tremella aurantialba*, the targets of the above active compounds were introduced into DAVID Database and the related biological process and pathways of *Tremella aurantialba* were obtained (Supplementary Table 3). As shown in Figure 3, the predicted biological processes and pathways are mainly involved with nervous, immune, endocrine, neoplasm, as well as cardiovascular diseases. The analysis results were consistent with the function of *Tremella aurantialba* and its active components reported.

**FIGURE 4:**
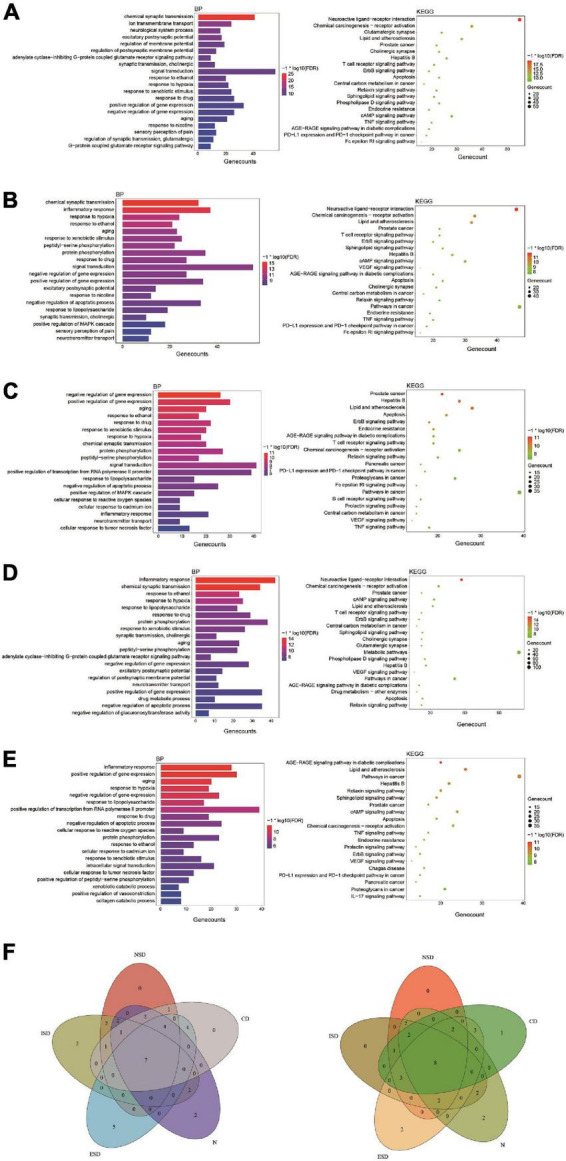
The enrichment analysis of overlapping targets from component-related and disease-related targets. **(A)** *Tremella aurantialba-*nervous system diseases; **(B)** *Tremella aurantialba-*immune system diseases; **(C)** *Tremella aurantialba-*endocrine system diseases; **(D)** *Tremella aurantialba-*neoplasm system diseases; **(E)** *Tremella aurantialba-*cardiovascular system diseases; **(F)** Venn diagram of the results of enrichment analysis of the intersection of *Tremella aurantialba*-five diseases. The results of GO-BP enrichment analysis are on the left and KEGG enrichment analysis are on the right.

Based on the enrichment analysis of components targets, the related targets of five disease above were, respectively, searched from NCBI, OMIM, CTD, TTD, GeneCards and DisGNet databases. After summarizing and repeating, 5521, 6506, 3414, 14810 and 2926 candidate genes related with nervous system, immune system, endocrine system, neoplasm and cardiovascular system were obtained, respectively (Supplementary Table 4). Then, the corresponding 282, 297, 205, 376 and 178 overlapping ones from component-related and disease-related targets were obtained with the National Genomics Data Center website (Figure 2 and Supplementary Table 5). Finally, further enrichment analysis was adopted to analyzed overlapping targets and the detailed results were shown in Figures 4A–E and Supplementary Table 6. It was indicated that *Tremella aurantialba* had potential effect on regulating overlapping targets and multiple pathways, and so on to be a latent multi-target, multi-pathway treatment for five diseases mentioned above. Moreover, the molecular mechanisms of *Tremella aurantialba* involved in the interactions between the five diseases were identified. For instance, neuroactive ligand-receptor interactions and chemical carcinogenesis-receptor activation are the first two most important metabolic pathways in the intervention of *Tremella aurantialba* on nervous system diseases, immune system diseases and neoplasm; AGE-RAGE signaling pathway in diabetic complications and lipid and atherosclerosis are the first two most important metabolic pathways for *Tremella aurantialba* to intervene in cardiovascular system diseases; Lipid and atherosclerosis and apoptosis are the first two most important pathways of *Tremella aurantialba* in the intervention of endocrine system diseases. Besides, chemical synaptic transmission and ion transmembrane transport are the first two most important biological processes of *Tremella aurantialba* in the intervention of nervous system diseases; Chemical synaptic transmission and inflammatory response are the first two most important biological processes of *Tremella aurantialba* in the intervention of immune system diseases, neoplasm and cardiovascular system diseases; Aging and response to ethanol are the first two most important biological processes in the treatment of endocrine system diseases by *Tremella aurantialba*.

Notably, for five diseases, GO enrichment analysis showed that the targets of *Tremella aurantialba* were always closely related to 7 biological processes, including response to xenobiotic stimulus, response to ethanol, response to drug, aging, response to hypoxia, positive regulation of gene expression and negative regulation of gene expression; KEGG pathway enrichment analysis indicated that the targets of *Tremella aurantialba* were always significantly enriched in 8 pathways, including AGE-RAGE signaling pathway in diabetic complications, lipid and atherosclerosis, hepatitis B, relaxin signaling pathway, prostate cancer, apoptosis, chemical carcinogenesis-receptor activation, ErbB signaling pathway (Figure 4F). Various evidences indicated that these key biological processes and metabolic pathways play important roles in five diseases, but how to block the occurrence and progression of diseases through these biological processes and pathways is still an important problem to be solved. Thus, this study offers a potential scheme to this thorny problem. This means that *Tremella aurantialba* may simultaneously intervene in five diseases of our concern through these key biological processes and metabolic pathways. Moreover, this work also provides new ideas for the treatment of current disease-related complications. Here, we discuss several important pathways of interest.

At present, AGE-RAGE signaling pathway in diabetic complications has been widely reported. The interaction between advanced glycation end products (AGEs) and its receptor RAGE can cause functional disorders of blood vessels and immune cells, changes in programmed cell death signals and so on (24). Endogenous AGEs are irreversibly formed and accumulated mainly under chronic hyperglycemic, thus most studies have emphasized the important role of AGE-RAGE signaling pathway in diabetic complications, such as diabetes-related cardiovascular disease (25), malignant neoplasms (26) and neuroinflammation (27). In addition, there are few reports that AGE-RAGE signaling pathway plays an important role in five diseases unrelated to diabetes. Only AGEs are considered to be a major cardiovascular risk factors unrelated to diabetes (28). Here, this work first provides theoretical support for *Tremella aurantialba* to hinder the occurrence and development of five diabetes-related diseases by inhibiting the AGE-RAGE signaling pathway that mediates diabetes complications.

The ErbB protein family is a family of four structurally related receptor tyrosine kinases, including EGFR1/ErbB1/HER1, EGFR2/ErbB2/HER2, EGFR3/ErbB3/HER3, and EGFR4/ErbB4/HER4. These receptors can form homodimers or heterodimers with each other, and dimers are required for signaling activity. So far, there are many evidences about the importance of ErbB signaling pathway in the development of cancer, nervous system diseases and cardiovascular diseases. For example, CARF promotes colorectal cancer stemness by activating the ErbB signaling pathway (29). ErbB activity amplifies signals through the core RAS pathway, thereby promoting KRAS-driven lung neoplasm (30). Head and neck squamous carcinomas can trigger the reprogramming and transformation of ErbB family members, which up-regulates ErbB3 at the transcriptional level and promotes neoplasm cell survival and growth (31). These studies mainly emphasized the function of ErbB1/2/3 in cancer while the role of ErbB4 in cancer remains controversial. Neuregulin-1 (NRG1) is a family of EGF-like factors that activates ErbB receptors. NRG1/ErbB signaling is a key regulatory pathway in the repair processes of pathologic central and peripheral nervous system (32). Xu et al. reported that NRG1/ErbB signaling pathway is one of the main target pathways of organophosphorus-induced spinal cord injury in mice, suggesting that NRG1/ErbB signaling pathway may play a functional role in the central nervous system, but not in the peripheral nervous system (33). Xu et al. found that tri-o-cresyl phosphate-induced hyperactivation of NRG1/ErbB signaling in Schwann cells might lead to the disturbance of neuropathy target esterase activity and degenerative pathology in spinal cord and sciatic nerve (34). Besides, vascular cells regulate cardiomyocyte survival and angiogenesis through NRG/ErbB signaling pathway, which has a protective paracrine effect on cardiac cells as well as vascular smooth muscle cells in the setting of an injury (35). Dang et al. first found that the effect of antipsychotic exposure on myocardial NRG1/ErbB signaling and activated NRG1/ErbB system in brain (36). In addition, excluding the deficiency of NRG1 signal transduction, NRG1/ErbB signaling pathway may be a promising therapeutic target for glucose intolerance, which may improve liver insulin sensitivity by inducing erBB1-ErBB3 dimerization, increasing ErbB3 phosphorylation, and thus play a hypoglycemic role. However, it has no effect on skeletal muscle insulin resistance, which is different from other studies and deserves further discussion (37). ErbB family also can induce immune cell infiltration and may influence the progression of skin melanoma through MDSC (38). In this work, the important relationship between ErbB signaling pathway and diseases of nervous system, immune system, endocrine system, neoplasm and cardiovascular system is proposed again, which is consistent with other reports. These results provide a basis for the intervention of five diseases through ErbB signaling pathway.

Relaxin (RLN) is a part of the insulin superfamily, including RLN1, RLN2 and RLN3. RLN2 acts as neuropeptides in the nervous system, as vasodilators and cardiac stimulants in the cardiovascular system, and as antifibrotic agents while the roles of RLN1 and RLN3 are unclear. Many of the effects of human gene-2 relaxin (H2 relaxin) are mediated by its homologous G protein-coupled receptor (GPCR) and relaxin family peptide receptor (RXFP1). For example, the activation of RXFP1 by rh-RLN2 can improve mast cell degranulation and neurological function by inhibiting NF-κB of PI3K-AKT/neoplasm necrosis factor-alpha-induced protein 3 (TNFAIP3) signaling pathway, which indicated that rh-RLN2 may be a promising therapeutic agent to reduce neuroinflammation and secondary brain injury in germinal matrix hemorrhage patients (39). RLN-2 produces endothelium- and NO-dependent relaxation of mouse mesenteric arteries by activation of RXFP1 coupled to Gi2-PI3K-eNOS pathway. Targeting vasodilatory Gi-protein-coupled RXFP1 pathways may provide promising opportunities for drug discovery in endothelial dysfunction and cardiometabolic disease (40). RLN can improve cardiac function, decrease the content of type I and type III Collagen in myocardial tissue, increase myocardial micro vessel density, and inhibit endothelial-mesenchymal transition-induced myocardial fibrosis through Notch-mediated signal transduction pathway (41). Besides, RLN can regulate a variety of cytokines and signaling pathways to treat cardiovascular disease and inflammation-related diseases (such as heart failure, diabetes) (42). Moreover, RLN2/RXFP1 signaling has recently been increasingly shown to mediate anti-apoptotic functions, angiogenesis and chemoresistance in cancer cells (43). RLN and its related receptor GPCR RXFP1 can form an autocrine signaling loop and promote the development and proliferation of ovarian cancer (44). RLN2/RXFP1 signaling induces cell invasion via the β-Catenin pathway in endometrial cancer (45). Here, the important role of the RLN signaling pathway in the five diseases is also emphasized, which makes it possible for *Tremella aurantialba* to fight disease through this pathway.

### Prediction of potential therapeutic targets

Protein-protein interaction (PPI) network was used to analyze the interactions between overlapping targets. Among the overlapped 282, 297, 205, 376 and 178 targets, a total of 598, 640, 446, 725 and 343 PPIs were obtained from STRING Database, respectively. By setting a degree value greater than 3, optimized PPI networks were further constructed using Cytoscape software (Supplementary Figure 6 and Supplementary Table 7). Furtherly, clusters from the PPI network were screened using MCODE modules and the results were shown in Supplementary Figure 7.

Finally, the corresponding 4, 6, 6, 6 and 5 key targets for *Tremella aurantialba* on five diseases (NSD, ISD, ESD, Neu and CSD) were obtained, respectively, by CytoHubba plugin analysis and the common core targets of *Tremella aurantialba* on five diseases were also obtained, such as AKT1, JUN and ESR1 (Table 2). The importance of these three targets in five diseases has also been highlighted. Due to their versatility, these three targets have been proposed by some researchers as potential therapeutic targets for some diseases (46).

**TABLE 2:** The key targets for *Tremella aurantialba* on five diseases.

Diseases	Key targets	Core key targets
NSD	AKT1, JUN, ESR1, RXRA	
ISD	RELA, AKT1, RXRA, ESR1, MAPK1, JUN	
ESD	ESR1, PIK3CA, AKT1, JUN, RELA, MAPK14	AKT1, JUN, ESR1
Neu	JUN, MAPK14, RELA, ESR1, RXRA, AKT1	
CSD	AKT1, JUN, ESR1, MAPK14, RELA	

### Screening of key active components of *Tremella aurantialba*

**FIGURE 5:**
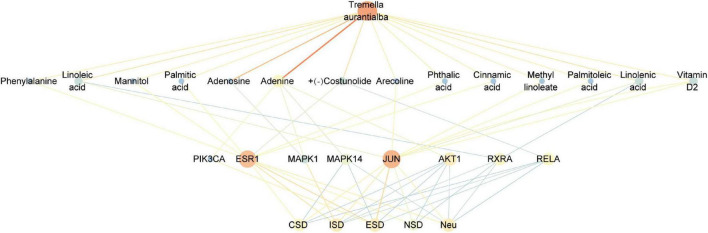
Components-targets-diseases network diagram of *Tremella aurantialba*.

**FIGURE 6:**
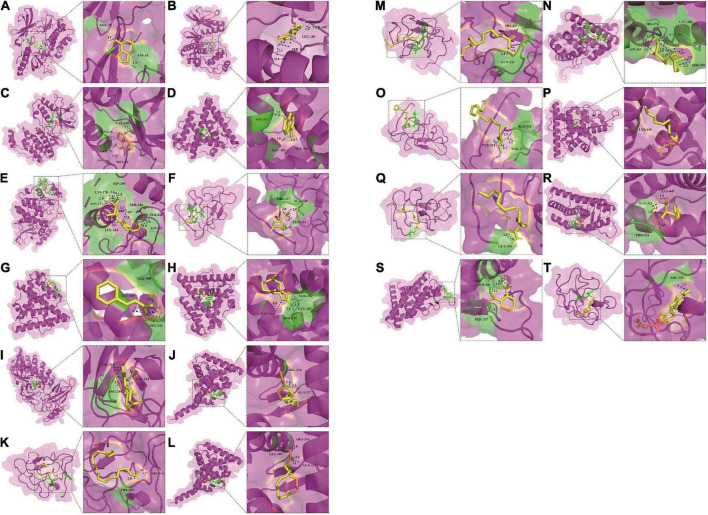
Molecular docking of 14 active compounds with key targets. **(A)** Three-dimensional binding mode of adenine and AKT1 (PDB: 3O96). **(B)** Three-dimensional binding mode of adenine and MAPK1 (PDB: 6SLG). **(C)** Three-dimensional binding mode of adenine and MAPK14 (PDB: 3PG3). **(D)** Three-dimensional binding mode of adenine and PIK3CA (PDB: 4A55). **(E)** Three-dimensional binding mode of adenosine and MAPK1 (PDB: 6SLG). **(F)** Three-dimensional binding mode of arecoline and JUN (PDB: 6OSN). **(G)** Three-dimensional binding mode of cinnamic acid and ESR1 (PDB: 5ACC). **(H)** Three-dimensional binding mode of costunolide and ESR1 (PDB: 5ACC). **(I)** Three-dimensional binding mode of costunolide and RELA (PDB: 1NFI). **(J)** Three-dimensional binding mode of linoleic acid and RXRA (PDB: 6JNO). **(K)** Three-dimensional binding mode of linoleic acid and JUN (PDB: 6OSN). **(L)** Three-dimensional binding mode of linolenic acid and RXRA (PDB: 6JNO). **(M)** Three-dimensional binding mode of linolenic acid and JUN (PDB: 6OSN). **(N)** Three-dimensional binding mode of mannitol and ESR1 (PDB: 5ACC). **(O)** Three-dimensional binding mode of methyl linoleate and JUN (PDB: 6OSN). **(P)** Three-dimensional binding mode of palmitic acid and ESR1 (PDB: 5ACC). **(Q)** Three-dimensional binding mode of palmitoleic acid and JUN (PDB: 6OSN). **(R)** Three-dimensional binding mode of phenylalanine and ESR1 (PDB: 5ACC). **(S)** Three-dimensional binding mode of phthalic acid and ESR1 (PDB: 5ACC). **(T)** Three-dimensional binding mode of vitamin D2 and JUN (PDB: 6OSN).

Fourteen active components, including adenine, palmitoleic acid, arecoline, linoleic acid, linolenic acid, methyl linoleate, vitamin D2, palmitic acid, phthalic acid, (+)-costunolide, phenylalanine, cinnamic acid, mannitol, adenosine, were obtained by reverse screening based on the above key targets. They were considered key active components that have the activity of exacerbating or fighting five diseases in this study. Except adenosine, which was only selected as a key compound for immune system, the other compounds were speculated to have potential effects on all five diseases. A visual network of key active compounds, key genes and five diseases were shown in Figure 5 and Supplementary Table 8. The further molecular docking study was adopted to predict the mode of the interaction between the above hub genes and the corresponding active compounds from *Tremella aurantialba*, based on the results of the network pharmacology analysis. Visual binding patterns showed good interaction between the receptor and ligand, especially linolenic acid and RXRA, with binding energy of –7.12, adenosine and MAPK1, with binding energy of –6.68, and vitamin D2 and JUN with binding energy of –6.63, suggesting that these compounds and targets may play a key role in the prevention and treatment of abdominal aortic aneurysms by *Tremella aurantialba* (Figure 6 and Supplementary Table 9). But the docking results were tentative, and only a speculation. Further *in vivo* and *in vitro* validation of the results is needed. In addition, according to the literature and other information, we further investigated the potential significant biological activities of these key compounds. Among them, the activities of most of the predicted compounds were consistent with those reported in the literature, and only a few compounds had different activity prediction results from those reported in the literature. For example, the fact that adenosine could also exert biological activity in other four diseases has been reported, but this study only obtained the potential of its effect on immune system diseases, which shows that the network pharmacology analysis method has certain limitations. It is not difficult to understand, because the network pharmacology analysis technology needs to be carried out on the basis of existing research, so it has a certain lag.

## Conclusion

As a famous medicinal and edible homologous fungus, the traditional pharmacological effects of *Tremella aurantialba* have been widely recorded in TCM books. However, there are few modern studies on the chemical components and pharmacological activities of *Tremella aurantialba*, which seriously hinder the development and utilization of *Tremella aurantialba*. Traditional Chinese medicine emphasizes the holistic concept and its drug use has the characteristics of multi-target and multi-function. However, for TCM with unclear chemical composition, blindly relying on the experience of ancestors will bring some safety risks to patients, and it is impossible to fully explore the pharmacological effects of *Tremella aurantialba*. In this study, UPLC-Q/TOF-MS was first used to investigate the chemical components of *Tremella aurantialba* and network pharmacological analysis was used to explore the potential pharmacological activities of *Tremella aurantialba*. Then, a total of 135 compounds were identified or tentatively characterized. Among them, the enrichment analysis of the targets of the active components of *Tremella aurantialba* suggests that *Tremella aurantialba* has potential pharmacological effects on nerve, immune, endocrine, neoplasm and cardiovascular diseases, which is consistent with the traditional Chinese medicine books and related literature reports. Next, the targets of five diseases were collected and collated, and the intersection with the targets of *Tremella aurantialba* components was obtained. Then, the main targets, biological processes and metabolic pathways of *Tremella aurantialba* for the intervention of five diseases were also obtained. Simultaneously, fourteen active components and their key targets were first screened by reverse screening to have potential therapeutic effect for nervous system, immune system, endocrine system, neoplasm system, as well as cardiovascular system diseases. Chinese medicine components are the material basis of efficacy. By extrapolating the active components acting on the key intersection targets, we hypothesized that these components might be the important material basis for the pharmacological activities of *Tremella aurantialba* in five diseases. In this regard, we used molecular docking technology combined with literature review to verify and investigate the above predicted results. Interestingly, we also identified key comorbid mechanisms for *Tremella aurantialba* to simultaneously intervene in five diseases. In this step, three core targets, seven biological processes and eight metabolic pathways were identified. These results provide reference for the treatment of some disease complications and the exploration of potential disease treatment targets. In conclusion, this study will provide new ideas for further understanding the pharmacological activities of dominant plant resources such as *Tremella aurantialba* and exploring key functional components (groups), and provide important research strategies for the full development and utilization of dominant plant resources. Of course, the prediction results need to be further verified *in vivo* and *in vitro*.

## Data availability statement

The original contributions presented in this study are included in the article/Supplementary material, further inquiries can be directed to the corresponding authors.

## Author contributions

YY: conceptualization, writing—original draft, and supervision. MW: methodology, investigation, and data curation. XG: data curation. XW, YL, and NC: validation. CF: supervision and writing—review and editing. PL: investigation, conceptualization, and funding acquisition. YZ: investigation, conceptualization, and writing—review and editing. All authors read and agreed to the published version of the manuscript.

## Footnotes

1
http://swissADME.ch/index.php

2
http://www.swisstargetprediction.ch/

3
https://bidd.group/NPASS/index.php

4
https://www.ncbi.nlm.nih.gov/

5
https://www.omim.org/

6
https://www.genecards.org/

7
http://db.idrblab.net/ttd

8
https://ctdbase.org/

9
https://www.disgenet.org/

10
https://www.uniprot.org/

11
https://ngdc.cncb.ac.cn/bit/venn

12
http://string-db.org

13
https://www.cytoscape.org/

14
https://david.ncifcrf.gov/

15
https://www.rcsb.org

16
https://pubchem.ncbi.nlm.nih.gov/

## References

[1] Yang L Li R Cao Y Li M Luo X Yang X Research on the scientific name and taxonomic status of *Tremella aurantialba*. *Edible Med Mushrooms.* (2020) 28:252–5.

[2] Zhang Y Lv P Ma J Chen N Guo H Chen Y *Antrodia cinnamomea* exerts an anti-hepatoma effect by targeting PI3K/AKT-mediated cell cycle progression *in vitro* and *in vivo*. *Acta Pharm Sin B.* (2022) 12:890–906. 10.1016/j.apsb.2021.07.010 35256953 PMC8897033

[3] Subbulakshmi M Dhanasekaran S Abirami S Kannan M Palaniappan R Venugopal D. Phylogenetic analysis and protective effects of thymol and its chromatographic fractions from a novel wild mushroom in combating oxidative stress. *Food Sci Hum Wellness.* (2021) 10:452–9. 10.1016/j.fshw.2021.04.007

[4] Dan A Hu Y Chen R Lin X Tian Y Wang S. Advances in research on chemical constituents and pharmacological effects of *Paecilomyces hepiali*. *Food Sci Hum Wellness.* (2021) 10:401–7. 10.1016/j.fshw.2021.04.002

[5] Hou R Liu X Wu X Zheng M Fu J. Therapeutic effect of natural melanin from edible fungus *Auricularia auricula* on alcohol-induced liver damage *in vitro* and *in vivo*. *Food Sci Hum Wellness.* (2021) 10:514–22. 10.1016/j.fshw.2021.04.014

[6] Han X Zhang J Liu Y Jia F Liu Z Xue B. Analysis of nutrition and volatile components of wild *Tremella aurantialba* in tibet under different drying methods. *Food Res Dev.* (2020) 41:49–55. 10.12161/j.issn.1005-6521.2020.13.008

[7] Li X Deng L Zhou Y Zhong L Zhao S Lei X Nutritional components comparison between *Tremella aurantialba, Tremella fucitormis* and *Auricularia aurricula*. *Food Res Dev.* (2021) 42:77–82. 10.12161/j.issn.1005-6521.2021.16.012

[8] Yuan Q Zhang X Ma M Long T Xiao C Zhang J Immunoenhancing glucuronoxylomannan from *Tremella aurantialba Bandoni et Zang* and its low-molecular-weight fractions by radical depolymerization: properties, structures and effects on macrophages. *Carbohydr Polym.* (2020) 238:116184. 10.1016/j.carbpol.2020.116184 32299559

[9] Du X Wang X Chen Y Tian S Lu S. Antioxidant activity and oxidative injury rehabilitation of chemically modified polysaccharide (TAPA1) from *Tremella aurantialba*. *Macromol Res.* (2018) 26:479–83. 10.1007/s13233-018-6078-0

[10] Dai C Huang X Lv R Zhang Z Sun J Aheto J. Analysis of volatile compounds of *Tremella aurantialba* fermentation via electronic nose and HS-SPME-GC-MS. *J Food Saf.* (2018) 38:e12555. 10.1111/jfs.12555

[11] Li Y. *Studies on chemical composition and antioxidantactivity in vitro from Tremella aurantialba fruiting bodies.* Changchun: Jilin Agricultural University (2016).

[12] Liu N Li J Guo C Guo Y. Effect of *Tremella aurantialba* lipid extracts on the penetration of evans blue through blood brain barrier. *Sci Technol Food Ind.* (2019) 40:62–6.

[13] Du X Zhang J Jia W. Antitum or and immunostimulating activities of the extracts from *Tremella aurantialba* fruting bodies *in vitro*. *Nat Prod Res Dev.* (2011) 23:351–5.

[14] Yuan Q Zhao L Li H Wei Z. Immunoenhancing glucuronoxylomannan from *Tremella aurantialba Bandoni et Zang* and its low-molecular-weight fractions by radical depolymerization: properties, structures and effects on macrophages. *Carbohydr Polym.* (2020) 238:116184. 10.1016/j.carbpol.2020.116184 32299559

[15] Wang D. *Study on extraction, isolation and purification of polysaccharide from tremella aurantialba and its antioxidant activity.* Liaocheng: Liaocheng University (2018).

[16] Cui L Liu Y Liu M Ren M Ahmed A Kang W. Identification of phytochemicals from *Lentinus edodes* and *Auricularia auricula* with UPLC-Q-exactive orbitrap MS. *J Future Foods.* (2022) 2:253–60. 10.1016/j.jfutfo.2022.06.006

[17] Yin Z Sun-Waterhouse D Wang J Ma C Waterhouse G Kang W. Polysaccharides from edible fungi *Pleurotus spp.*: advances and perspectives. *J Future Foods.* (2021) 1:128–40. 10.1016/j.jfutfo.2022.01.002

[18] Yin Z Liang Z Li C Wang J Ma C Kang W. Immunomodulatory effects of polysaccharides from edible fungus: a review. *Food Sci Hum Wellness.* (2021) 10:393–400. 10.1016/j.fshw.2021.04.001

[19] Zhang Y Ma A Xi H Chen N Wang R Yang C *Antrodia cinnamomea* ameliorates neointimal formation by inhibiting inflammatory cell infiltration through downregulation of adhesion molecule expression *in vitro* and *in vivo*. *Food Sci Hum Wellness.* (2021) 10:421–30. 10.1016/j.fshw.2021.04.004

[20] Pérez-Navarro J Da Ros A Masuero D Izquierdo-Cañas P Hermosín-Gutiérrez I Gómez-Alonso S LC-MS/MS analysis of free fatty acid composition and other lipids in skins and seeds of *Vitis vinifera grape cultivars*. *Food Res Int.* (2019) 125:108556. 10.1016/j.foodres.2019.108556 31554044

[21] Aliabadi M Karami-Osboo R Kobarfard F Jahani R Nabi M Yazdanpanah H Detection of lime juice adulteration by simultaneous determination of main organic acids using liquid chromatography-tandem mass spectrometry. *J Food Compost Anal.* (2022) 105:104223. 10.1016/j.jfca.2021.104223

[22] Kubica P Kot-Wasik A Wasik A Namieśnik J Landowski P. Modern approach for determination of lactulose, mannitol and sucrose in human urine using HPLC-MS/MS for the studies of intestinal and upper digestive tract permeability. *J Chromatogr B.* (2012) 907:34–40. 10.1016/j.jchromb.2012.08.031 22985725

[23] Caprioli G Sagratini G Vittori S Torregiani E. Optimization of an extraction procedure for the simultaneous quantification of riboflavin, nicotinamide and nicotinic acid in anchovies (*Engraulis enrasicolus*) by high-performance liquid chromatography-tandem mass spectrometry. *J Food Compost Anal.* (2018) 66:23–9. 10.1016/j.jfca.2017.11.004

[24] Waghela B Vaidya F Ranjan K Chhipa A Tiwari B Pathak C. AGE-RAGE synergy influences programmed cell death signaling to promote cancer. *Mol Cell Biochem.* (2021) 476:585–98. 10.1007/s11010-020-03928-y 33025314

[25] Burr S Dorroh C Stewart J Jr. Rap1a activity elevated the impact of endogenous AGEs in diabetic collagen to stimulate increased myofibroblast transition and oxidative stress. *Int J Mol Sci.* (2022) 9:4480. 10.3390/ijms23094480 35562872 PMC9101126

[26] Pan S Guan Y Ma Y Cui Q Tang Z Li J Advanced glycation end products correlate with breast cancer metastasis by activating RAGE TLR4 signaling. *BMJ Open Diabetes Res Care.* (2022) 10:e2697. 10.1136/bmjdrc-2021-002697 35346972 PMC8961114

[27] Yu X Zhang D Xiao C Zhou Y Li X Wang L P-Coumaric acid reverses depression-like behavior and memory deficit via inhibiting AGE-RAGE-mediated neuroinflammation. *Cells.* (2022) 11:1594. 10.3390/cells11101594 35626632 PMC9139330

[28] Kosmopoulos M Drekolias D Zavras P Piperi C Papavassiliou A. Impact of advanced glycation end products (AGEs) signaling in coronary artery disease. *Biochim Biophys Acta (BBA) Mol Basis Dis.* (2019) 1865:611–9. 10.1016/j.bbadis.2019.01.006 30611860

[29] Dong W Cao Z Pang Y Feng T Tian H. CARF, as an oncogene, promotes colorectal cancer stemness by activating ERBB signaling pathway. *Onco Targets Ther.* (2019) 12:9041–51. 10.2147/OTT.S225733 31802911 PMC6830361

[30] Kruspig B Monteverde T Neidler S Hock A Kerr E Nixon C The ERBB network facilitates KRAS-driven lung tumorigenesis. *Sci Transl Med.* (2018) 10:eaao2565. 10.1126/scitranslmed.aao2565 PMC6881183 29925636

[31] Humtsoe J Pham E Louie R Chan D Kramer R. ErbB3 upregulation by the HNSCC 3D microenvironment modulates cell survival and growth. *Oncogene.* (2016) 35:1554–64. 10.1038/onc.2015.220 26073080

[32] Kataria H Alizadeh A Karimi-Abdolrezaee S. Neuregulin-1 ErbB network an emerging modulator of nervous system injury and repair. *Prog Neurobiol.* (2019) 180:101643. 10.1016/j.pneurobio.2019.101643 31229498

[33] Xu H Sun Y Sun Y Wu Y Xu M Chen L Lapatinib alleviates TOCP-induced axonal damage in the spinal cord of mouse. *Neuropharmacology.* (2021) 189:108535. 10.1016/j.neuropharm.2021.108535 33766630

[34] Xu H Wang P Sun Y Xu M Zhu L Wu Y. Activation of neuregulin 1 ErbB signaling Is involved in the development of TOCP-induced delayed neuropathy. *Front Mol Neurosci.* (2018) 11:129. 10.3389/fnmol.2018.00129 29740279 PMC5925568

[35] Hedhli N Kalinowski A Russell K. Cardiovascular effects of neuregulin-1 ErbB signaling role in vascular signaling and angiogenesis. *Curr Pharm Des.* (2014) 20:4899–905. 10.2174/1381612819666131125151058 24283954

[36] Dang R Guo Y Cai H Yang R Liang D Lv C Effects of prolonged antipsychotic administration on neuregulin-1 ErbB signaling in rat prefrontal cortex and myocardium implications for the therapeutic action and cardiac adverse effect. *J Toxicol.* (2016) 41:303–9. 10.2131/jts.41.303 26961615

[37] Caillaud K Boisseau N Ennequin G Chavanelle V Etienne M Li X Neuregulin 1 improves glucose tolerance in adult and old rats. *Diabetes Metab.* (2016) 42:96–104. 10.1016/j.diabet.2015.08.003 26404652

[38] Liu S Geng R Lin E Zhao P Chen Y. ERbB family can induce immune cell infiltration and may influence the progression of skin melanoma through MDSC. *Front Genet.* (2021) 12:602160. 10.3389/fgene.2021.602160 33732282 PMC7957073

[39] Li P Zhao G Chen F Ding Y Wang T Liu S Rh-relaxin-2 attenuates degranulation of mast cells by inhibiting NF-κB through PI3K-AKT TNFAIP3 pathway in an experimental germinal matrix hemorrhage rat model. *J Neuroinflammation.* (2020) 17:250. 10.1186/s12974-020-01926-x 32859236 PMC7455905

[40] Lian X Beer-Hammer S König G Kostenis E Nürnberg B Gollasch M. RXFP1 receptor activation by relaxin-2 induces vascular relaxation in mice via a Gαi2-Protein/PI3K/γ/Nitric oxide-coupled pathway. *Front Physiol.* (2018) 9:1234. 10.3389/fphys.2018.01234 30233409 PMC6131674

[41] Zhou X. Relaxin inhibits cardiac fibrosis and endothelial-mesenchymal transition via the Notch pathway. *Drug Des Devel Ther.* (2015) 9:4599–611. 10.2147/DDDT.S85399 26316699 PMC4541540

[42] Martin B Gabris-Weber B Reddy R Romero G Chattopadhyay A Salama G. Relaxin reverses inflammatory and immune signals in aged hearts. *PLoS One.* (2018) 13:e190935. 10.1371/journal.pone.0190935 29346407 PMC5773192

[43] Rizvi S Gores G. The two faces of relaxin in cancer: antitumor or protumor? *Hepatology.* (2021) 71:1117–9. 10.1002/hep.30998 31630431 PMC7089805

[44] Burston H Kent O Communal L Udaskin M Sun R Brown K Inhibition of relaxin autocrine signaling confers therapeutic vulnerability in ovarian cancer. *J Clin Invest.* (2021) 13:e142677. 10.1172/JCI142677 33561012 PMC8011889

[45] Fue M Miki Y Takagi K Hashimoto C Yaegashi N Suzuki T Relaxin 2 RXFP1 signaling induces cell invasion via the β-catenin pathway in endometrial cancer. *Int J Mol Sci.* (2018) 19:2438. 10.3390/ijms19082438 30126180 PMC6121407

[46] Novoszel P Holcmann M Stulnig G De Sa F Zyulina V Borek I Psoriatic skin inflammation is promoted by c-Jun/AP-1-dependent CCL2 and IL-23 expression in dendritic cells. *EMBO Mol Med.* (2021) 13:e12409. 10.15252/emmm.202012409 33724710 PMC8033525

