# Supplementary material for: Evaluation of pharmacological activities and active components in Tremella aurantialba by instrumental and virtual analyses
Source: Front Nutr. 2022 Dec 7;9:1083581. doi: 10.3389/fnut.2022.1083581 (PMC9767953; doi:10.3389/fnut.2022.1083581)
Supplement: Supplementary file 2 [file Data_Sheet_2.DOCX]

**Supporting Information for Original article**

**Evaluation of pharmacological activities and active components in *Tremella aurantialba* by instrumental and virtual analyses**

Yonghuan Yan ^a, b^, Mengtian Wang ^a, b^, Xiaoruo Gan ^c^, Xu Wang ^b^, Chenghao Fu ^c^, Yuemin Li ^c^, Ning Chen ^c^, Pin Lv ^c, *^, Yan Zhang ^a, b, *^

^a^ School of Forensic Medicine, Hebei Key Laboratory of Forensic Medicine, Hebei Medical University, Shijiazhuang, 050017, China.

^b^ Hebei Food Inspection and Research Institute, Hebei Food Safety Key Laboratory, Hebei Province, Shijiazhuang, 050227, China.

^c^ Department of Cell Biology, Cardiovascular Medical Science Center, Key Laboratory of Neural and Vascular Biology of Ministry of Education, Hebei Medical University, Shijiazhuang 050017, China.

* Corresponding author at: Department of Sanitary Inspection, School of Forensic Medicine, Hebei Medical University, Shijiazhuang, 050017, China; Hebei Food Inspection and Research Institute, Hebei Food Safety Key Laboratory, Shijiazhuang, China. E-mail addresses: snowwinglv@126.com (Y. Zhang)

* Corresponding author at: Department of Cell Biology, Cardiovascular Medical Science Center, Key Laboratory of Neural and Vascular Biology of Ministry of Education, Hebei Medical University, Shijiazhuang 050017, China. E-mail addresses: lvpin@hebmu.edu.cn (P. Lv)


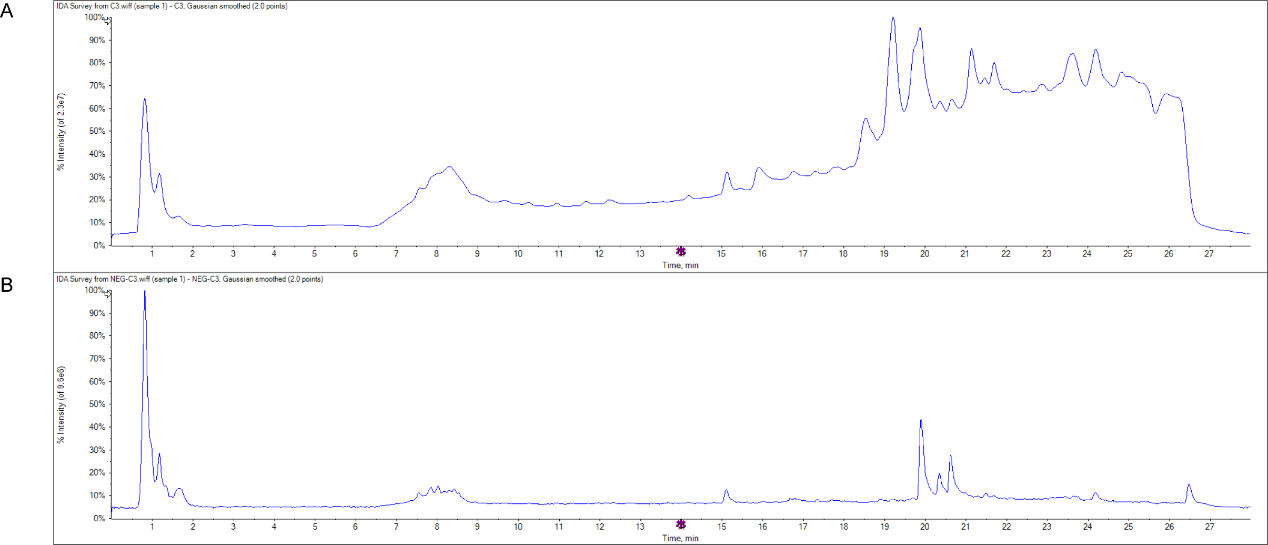


**Fig. S1. The total ion chromatograms of *Tremella aurantialba* in positive (A) and negative (B) ion modes.**


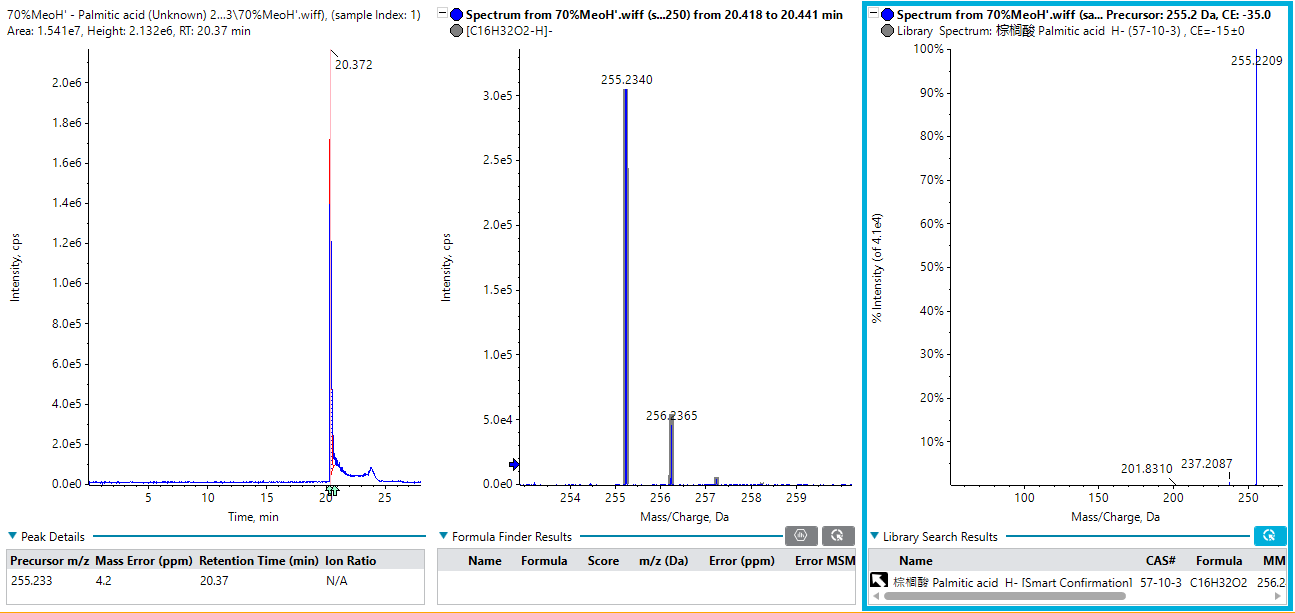


**Fig. S2. The mass spectra and fragment information and Sciex OS screening interface of palmitic acid in negative mode.**


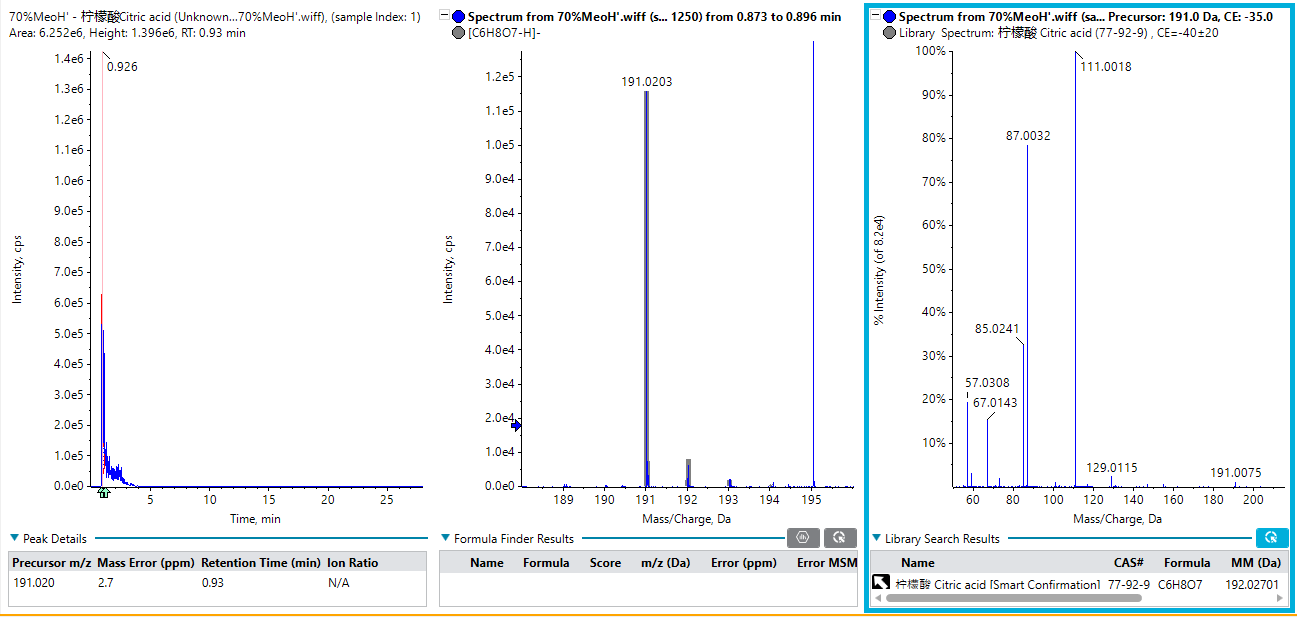


**Fig. S3. The mass spectra, fragment information and Sciex OS screening interface of citric acid in negative mode.**


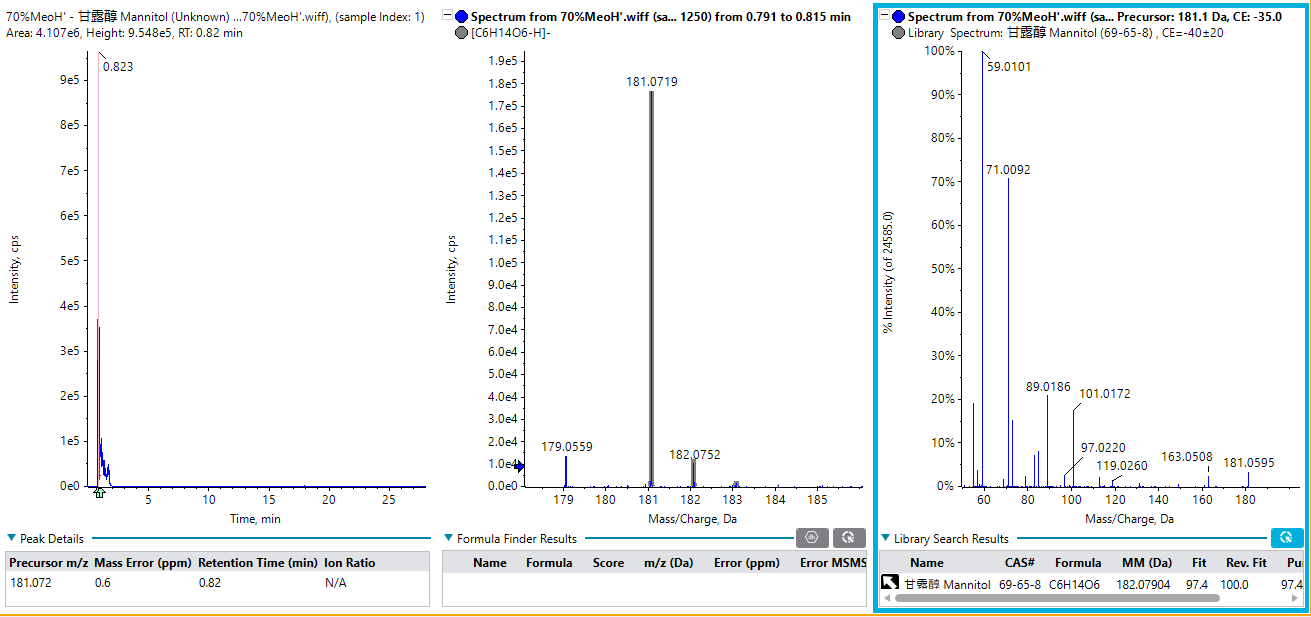


**Fig. S4. The mass spectra and fragment information and Sciex OS screening interface of mannitol in negative mode.**


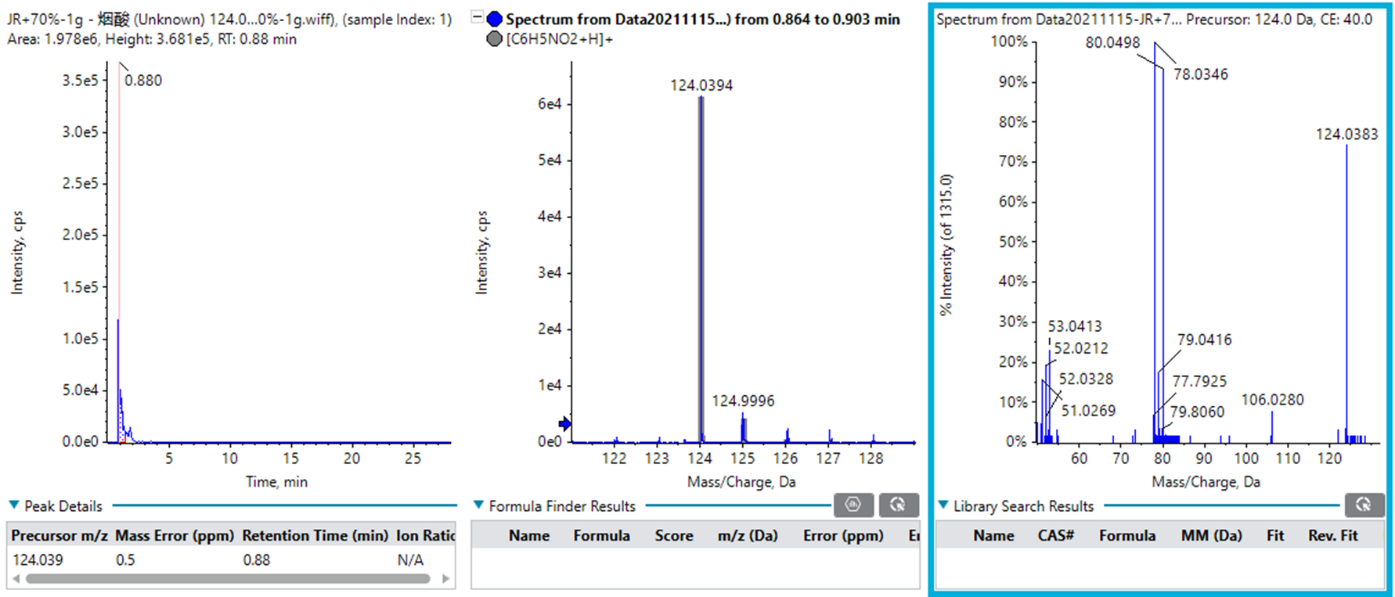


**Fig. S5. The mass spectra, fragment information and Sciex OS screening interface of nicotinic acid in positive mode.**


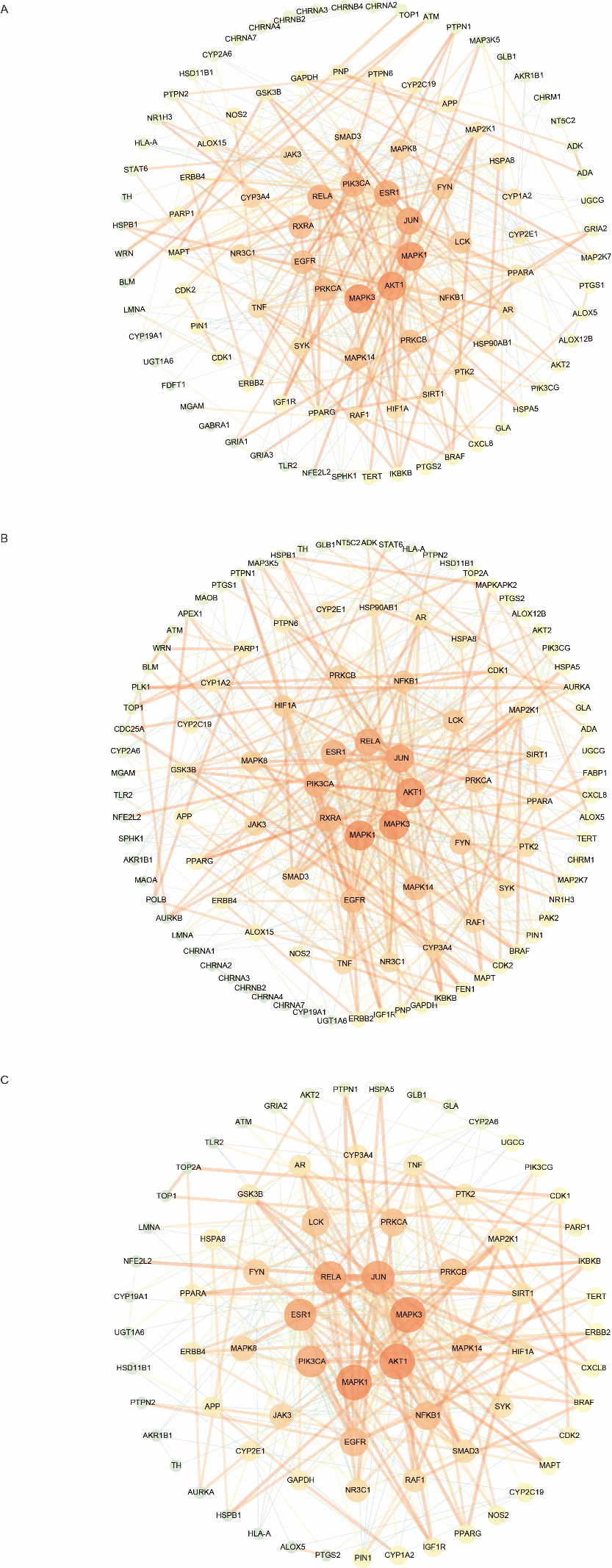

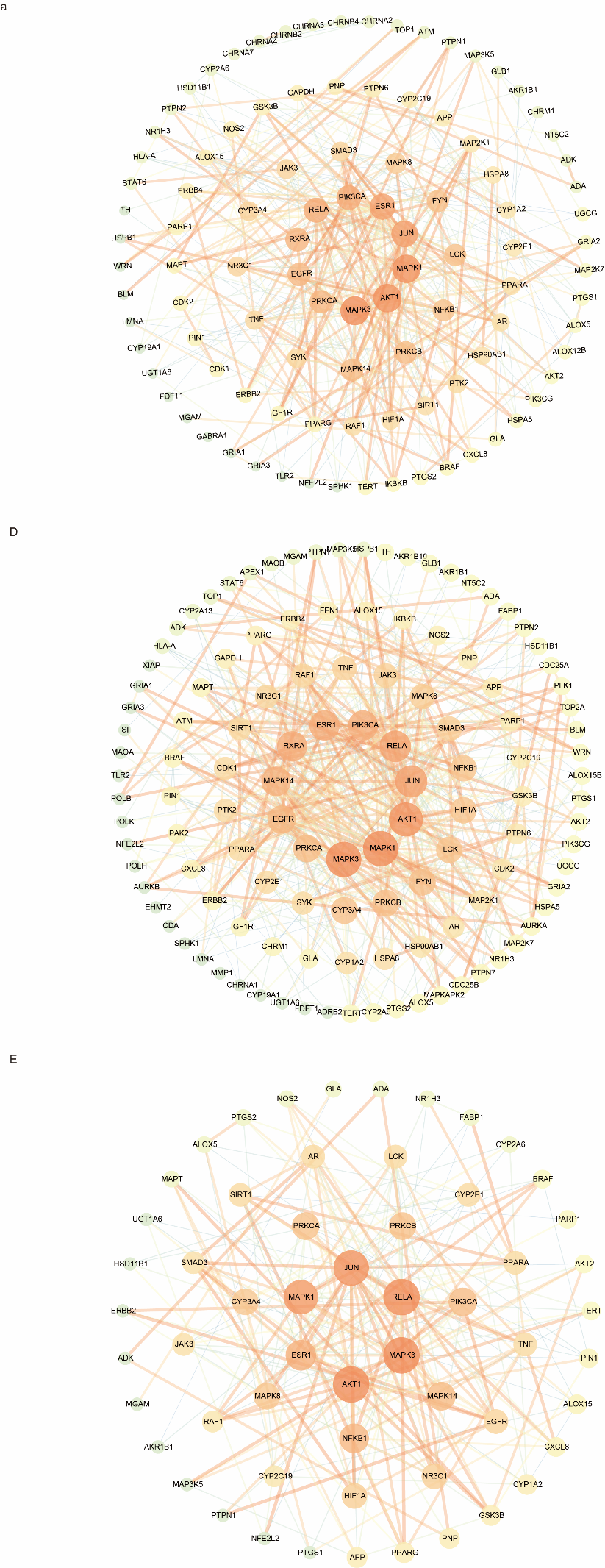


**Fig. S6. The PPI network of overlapping targets.** (A) *Tremella aurantialba-*nervous system diseases; (B) *Tremella aurantialba-*immune system diseases; (C) *Tremella aurantialba-*endocrine system diseases; (D) *Tremella aurantialba-* neoplasm system diseases; (E) *Tremella aurantialba-*cardiovascular system diseases.


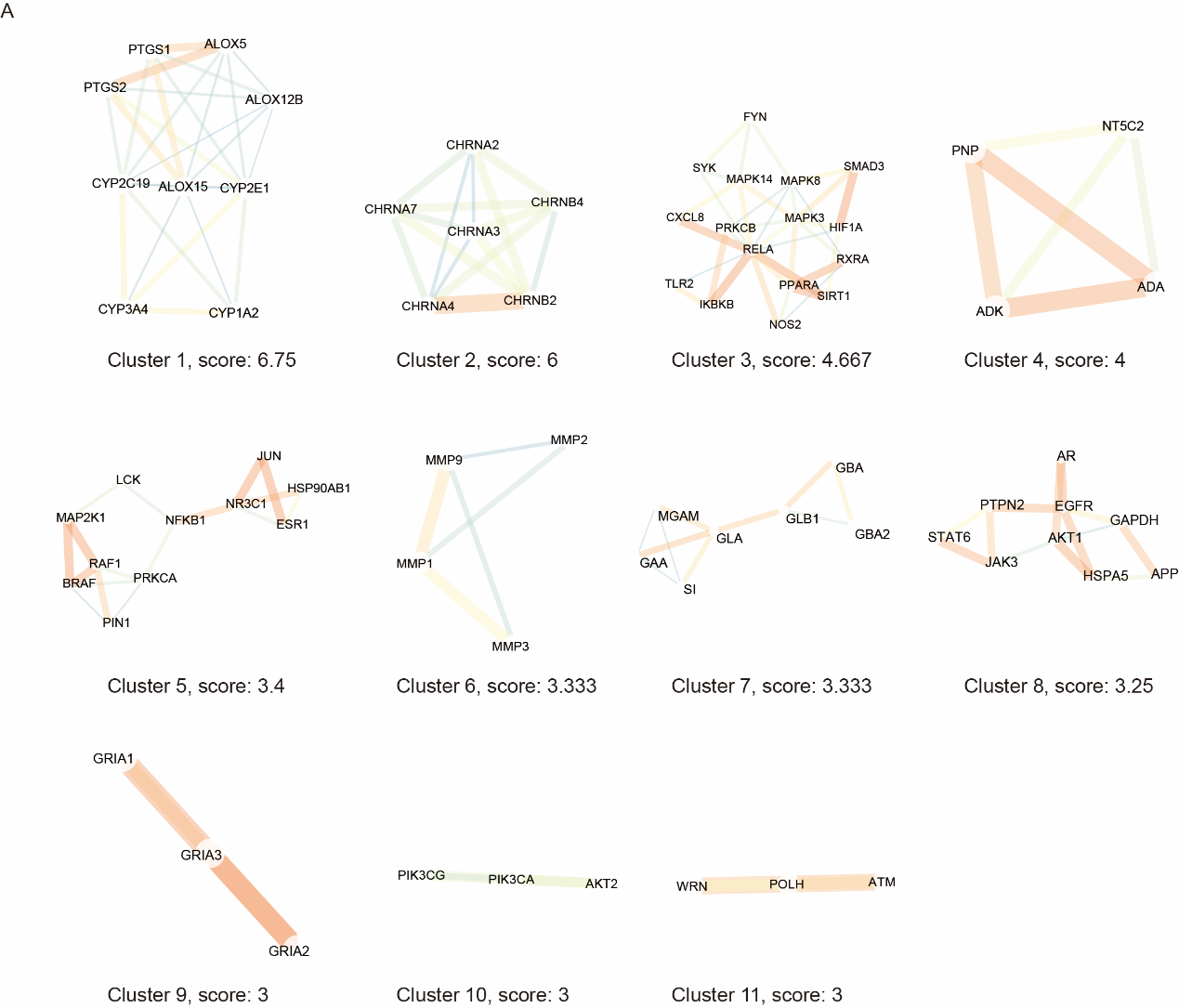


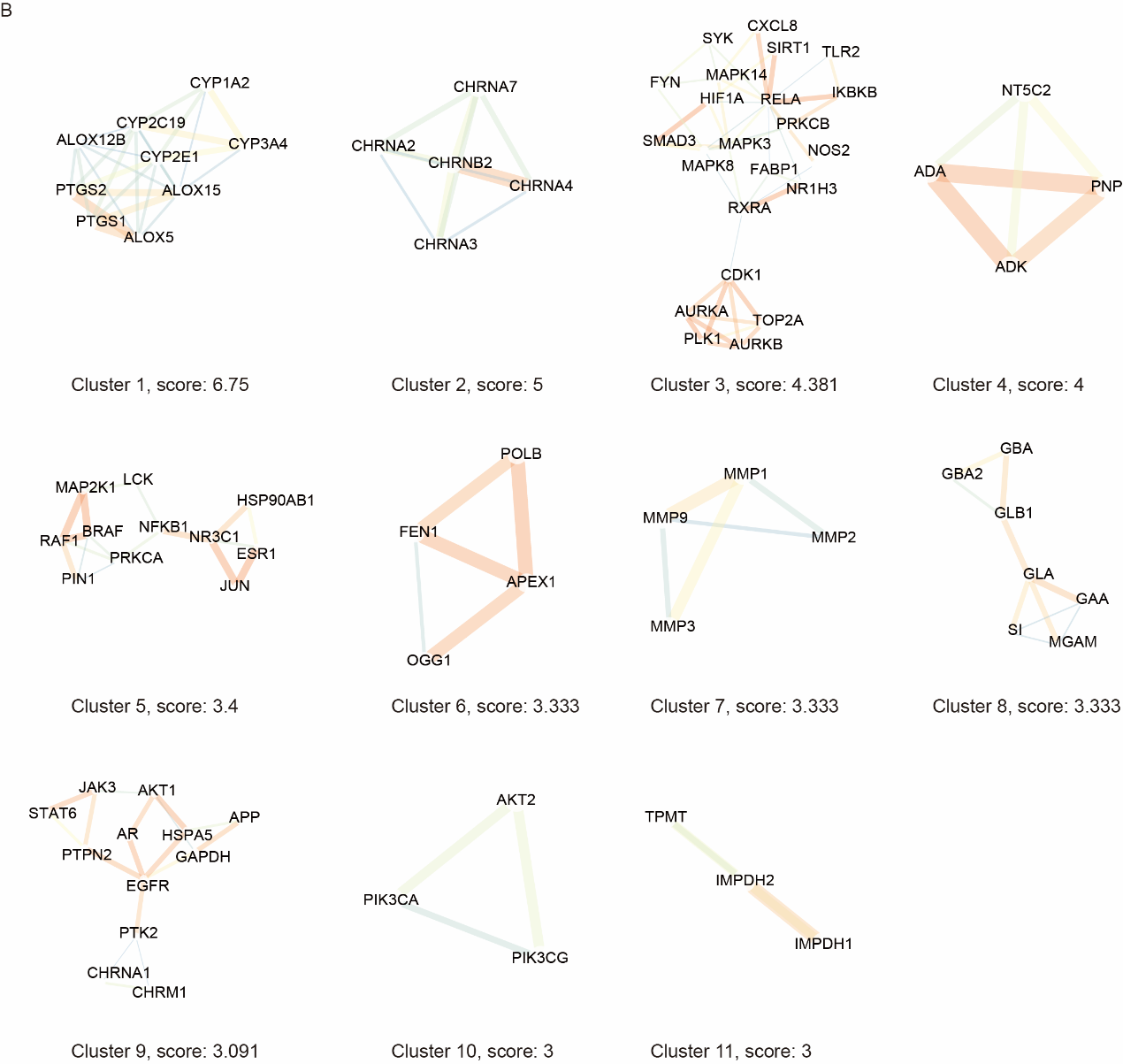


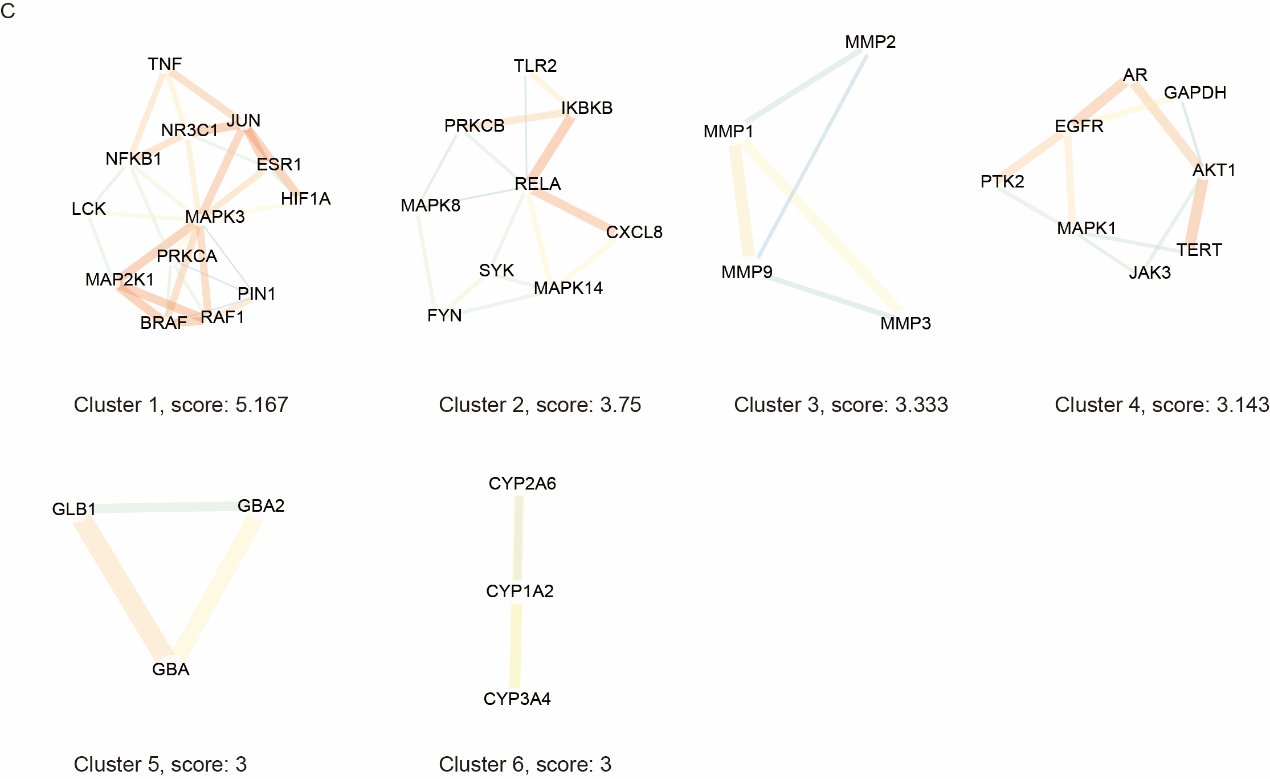

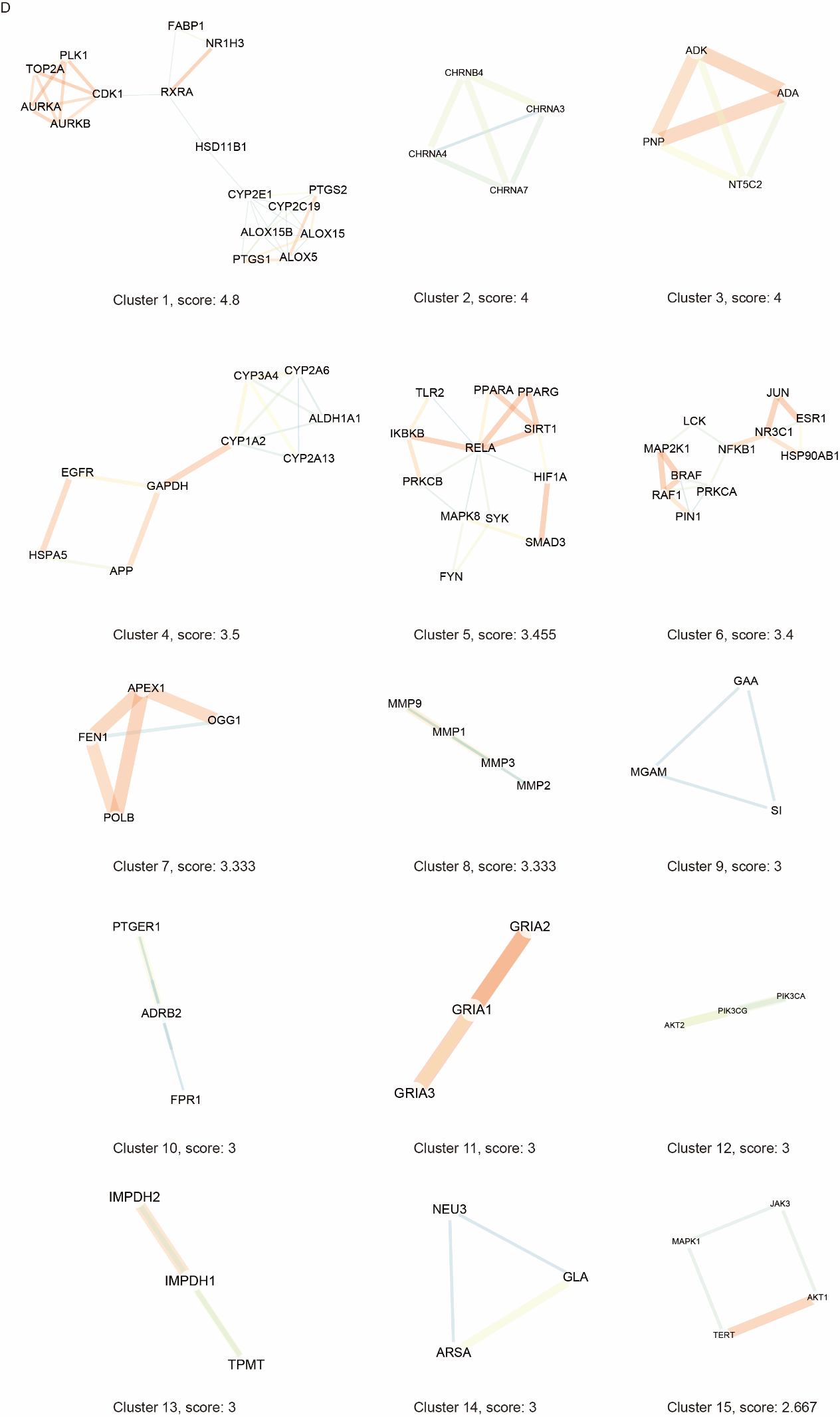

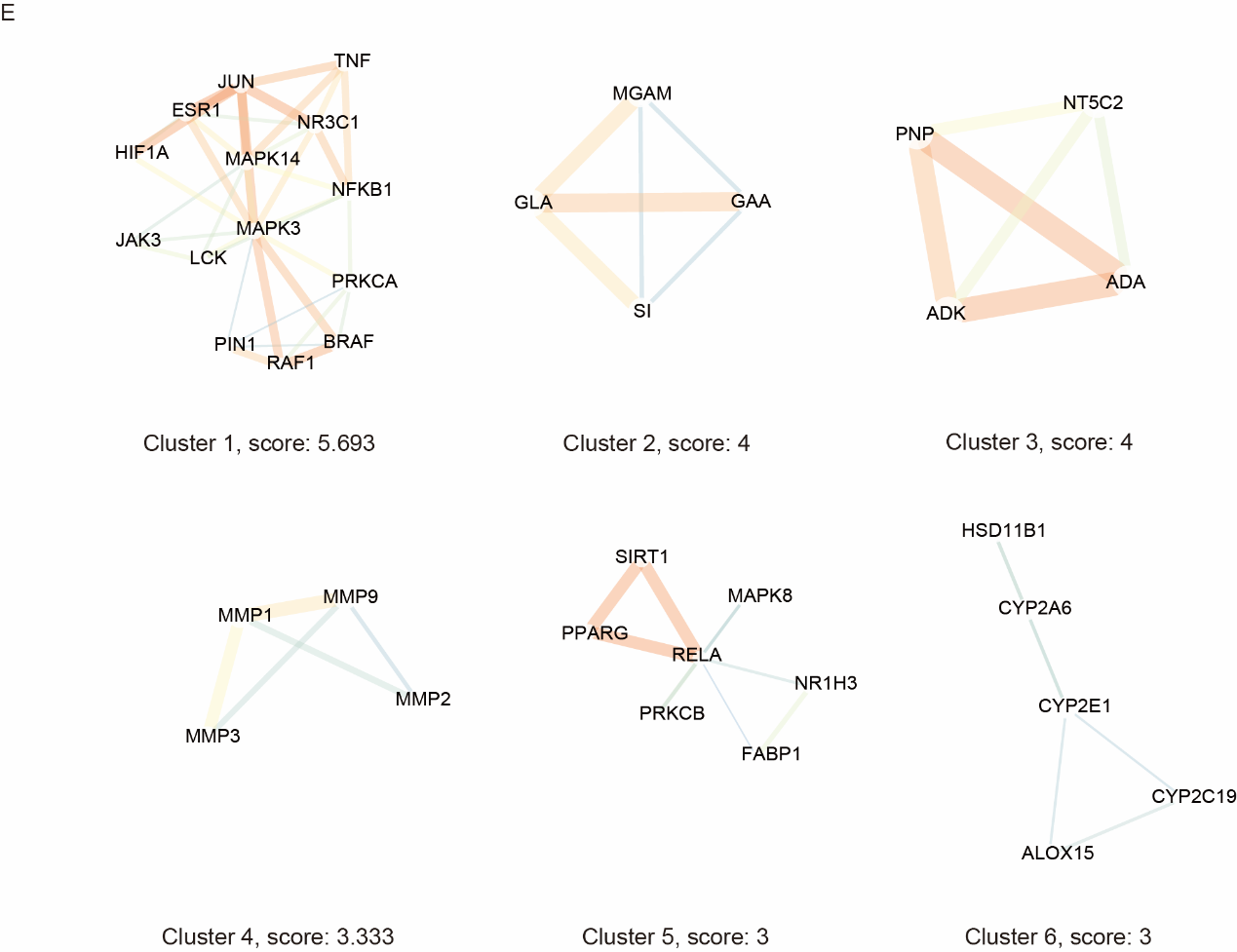


**Fig. S7. MCODE analysis of overlapping targets of components-diseases.** (A) *Tremella aurantialba-*nervous system diseases; (B) *Tremella aurantialba-*immune system diseases; (C) *Tremella aurantialba-*endocrine system diseases; (D) *Tremella aurantialba-*neoplasm system diseases; (E) *Tremella aurantialba-*cardiovascular system diseases.

**Table S2.** Reported pharmacological activities of the key active components of *Tremella aurantialba*.

| No. | Key active components | Reported pharmacological activity |
| --- | --- | --- |
| 1 | Adenine | Adenine is a key alkaloid that plays an important role in various life processes, including genetic information storage, energy transfer and cell signaling. Adenine has been successfully used in the prevention and treatment of acute granulocytopenia and leukopenia caused by various causes, especially leukopenia caused by chemical and radiation therapy for cancer and benzene poisoning (Hartmann & Getoff, 2006; Ohyama & Yamada, 1970; Tello Ortiz & Contreras Taboada, 1959).  Nervous system: Adenine activates AMP-activated protein kinase (AMPK), which is used to prevent or treat conditions or diseases improved by AMPK in mammals, such as wounds, ulcers, burns, scars, alzheimer's disease and other neurodegenerative diseases of the central nervous system (CHEN and CHIO, et al., 2015); Adenine has neuroprotective effect on the survival of purkinje cells from primary culture of rat cerebellum (Watanabe, Yoshimi & Ikekita, 2003).  Immune system: Adenine has anti-inflammatory effects in vitro, which may involve the increase of AMP in THP-1 cells catalyzed by adenine phosphoribosyltransferase and the activation of AMPK stimulated by adenine (Wu, Chen, Lin, Young, Wang & Chen, 2019); Adenine attenuates lPS-induced immune cell inflammation (Silwal, Lim, Heo, Park, Namgung & Park, 2018); Adenine can enhance the osteogenic effect of osteoblast MG-63 cells in both normal and inflammatory conditions, which is expected to be used in the treatment of osteoporosis and other inflammatory bone diseases (Chen, Chu, Tsuang, Wu, Kuo & Kuo, 2020); Adenine inhibits TNF-α signaling in intestinal epithelial cells and reduces mucosal inflammation in mouse models of colitis induced by sodium glucan sulfate, which is expected to be used for prevention of enteritis (Fukuda, Majumder, Zhang, Turner, Matsui & Mine, 2016); Adenine can treat allergic diseases by inhibiting Fc Epsilon RI signaling in mast cells (Silwal et al., 2015)；Adenine can induce chronic renal failure in small and rats (Nemmar et al., 2016), hyperphosphatemia (Terai, Mizukami & Okada, 2008) , and reproductive insufficiency in small and rats (Adachi, Sasagawa & Nakada, 1993), and cause acute reversible renal tubular necrosis, and has immunosuppressive effect on mouse renal tissue (Chalmers, Rotstein, Mohan, Marshall & Coleman, 1985).  Endocrine system: Adenine can induce AMPK activation and interfere with AMPK-mediated glucose metabolism, providing reference for the treatment of insulin resistance and metabolic dysfunction (Young et al., 2015); Adenine can induce selective apoptosis of chronically infected HIV cells in vitro (Hirasawa, Yoshida, Fujinami, Sohma & Watanabe, 2000).  Neoplasm: Adenine has potential anti-proliferation and anti-metastasis abilities on tumor cells, and inhibits the invasion potential of Human colorectal cancer cells DLD-1 through AMPK/FAK axis (Huang et al., 2021); Adenine inhibits hepatocellular carcinoma cell growth through AMPK-mediated S phase stagnation and apoptotic cascade (Su, Huang, Chen, Lin & Kao, 2020); Adenine inhibits the growth of colon cancer cells through AMPK - mediated autophagy (Lai, Wei, Hung & Lin, 2019); Adenine induces cell cycle arrest and autophagy in K562 cells through amP-activated protein kinase signaling, thus contributing to the treatment of chronic myelogenous leukemia (Chen, Lin, Lin, Cheng, Chen & Kao, 2017).  Cardiovascular system: Adenine protects H9C2 rat myocardium damaged by hypoxia and reoxygenation by activating PPAR δ (Leu et al., 2022); Adenine activates AMPK and alleviates TNF-α - induced inflammatory responses in human umbilical vein endothelial cells (Cheng et al., 2015); The vascular relaxant adenine inhibits Ca2+ contractile signaling by activating the AdeR/ PKA axis in aortic vascular smooth muscle cells (Fukuda et al., 2016).  Others: Hair growth promoter (Chen and Jin, 2015); Adenine can promote collagen synthesis and inhibit collagenase activity, prevent and improve skin wrinkles (CHOUNG and EOM, et al., 2005); Antiviral activity (HIRASAWA and FUJINAMI, et al., 2000); Adenine prevents scarring formation during wound healing and inhibits fibroblast proliferation during wound healing, treating metabolic syndromes such as diabetes (Chen and Kuo, et al., 2019); Adenine can induce damage of liver and kidney function and result in spermatogenesis dysfunction of testis in male rats (Yu and Guo, et al., 2021); Adenine can reduce iron overload in mice by cAMP/PKA mediated hepatic iron modulin, thus treating hereditary chromosomia (Zhang et al., 2018); Adenine can induce meiosis arrest of mouse oocyte (Li, Wang & Fan, 1994); Adenine (200 mg/kg) inhibited the motor activity of mice (Akintonwa & Auditore, 1978); Adenine induces increased hepatic glucose release (Kaldor, Rihan, Nichols & Butterfield, 1964). |
| 2 | Costunolide | Nervous system: Costunolide has significant neuroprotective activity and can protect PC12 cells from oxygen-glucose deprivation/reperfusion injury by blocking calcium channels and inhibiting mitochondria mediated apoptosis (Meng, Ma, Meng, Li, Zhu & Zhao, 2021); Costunolide alleviates intestinal dysfunction and depressive behavior in stress-induced irritable bowel syndrome mice by inhibiting mast cell activation in the colon and regulating serotonin metabolism in the central nervous system (Li et al., 2021).  Immune system: Costunolide has anti-inflammatory and immunomodulatory activities and can improve colitis by specifically inhibiting HIF1 α/ glycoly-mediated Th17 differentiation, laying the foundation for a new perspective of immunomodulatory treatment of colitis (Lv et al., 2021); Costunolide can inhibit THE differentiation of CD4 (+) T cells by inhibiting ERIC and P38 activities, and can be an effective therapeutic agent for T cell-mediated immune diseases (Park, Song, Kim, Park & Kim, 2016).  Endocrine system: Costunolide has hypoglycemic and hypidemic activities and can stimulate the secretion of insulin by inhibiting the expression of nitric oxide synthase, which is a potential drug in the treatment of diabetes (Eliza, Daisy, Ignacimuthu & Duraipandiyan, 2009).  Neoplasm: Costunolide has been reported to have significant anti-tumor activities against skin cancer, breast cancer, colorectal cancer, ovarian cancer and other cancers, such as activating p53 to inhibit glutaminase 1, blocking glutamine dissolution and inhibiting proliferation of colorectal cancer cells (Hu, Liu & Yao, 2018); Costunolide inhibits skin cancer by inducing apoptosis and blocking cell proliferation (Lee, Cho & Lim, 2021).  Cardiovascular system: Costunolide inhibits angiogenesis by blocking vascular growth factor signaling pathways (Jeong et al., 2002); Costunolide inhibits inflammatory angiogenesis in a subcutaneous mouse sponge model (Sarawati, Alhaider & Abdelgadir, 2018).  Others: Costunolide protects against alcohol-induced liver damage by regulating gut microbiota, oxidative stress, and reducing inflammation in vivo and in vitro (Meng et al., 2022); Costunolide inhibits pulmonary fibrosis by regulating NF-KB and TGF-β (1)/Smad (2)/Nrf (2)-NOX4 signaling pathways (Liu et al., 2019)。 |
| 3 | Vitamin D2 | The receptor for vitamin D2 (VD2), VDR, is a nuclear hormone receptor and transcription factor that is widely expressed in various tissues such as liver, fat and intestine. In the past, VD2 was thought to be a maturation regulator of calcium and phosphate metabolism, promoting healthy bone tissue growth (Charoenngam, Shirvani & Holick, 2019; Drori et al., 2016; Prietl, Treiber, Pieber & Amrein, 2013); In recent years, VD2 has also been reported to have neuroprotective activities, significant anti-inflammatory, immunomodulatory activities, anti-thrombosis, maintenance of endothelial cell function, heart protection, anti-tumor, anti-infection, anti-virus, anti-aging activities and significant effects on oral homeostasis (Caccamo, Ricca, Currò & Ientile, 2018; Mohammad, Mishra & Ashraf, 2019; Ślebioda, Szponar & Dorocka-Bobkowska, 2016).  Nervous system: Vitamin D has neurotrophic and neuroprotective properties, but whether it promotes cognitive impairment and brain atrophy remains controversial (Zelzer et al., 2021).  Immune system and endocrine system: Vitamin D can improve hyperglycemia, dyslipidemia, REDOX imbalance, inflammation and apoptosis, and protect the myocardium of diabetic rats from acute myocardial infarction (EI Agaty, 2019); Adequate levels of vitamin D can reduce the prevalence of fasting blood glucose and type 2 diabetes, especially in women with high testosterone levels (Wang et al., 2021).  Cancer: Vitamin D can induce cell cycle stagnation and apoptosis, and prevent the spread and metastasis of cancer (Petrou, Mamais, Lavranos, Tzanetakou & Chrysostomou, 2018). However, other studies have shown that circulating vitamin D levels are not associated with cancer (Muller et al., 2018).  Cardiovascular system: Vitamin D induces calcification of blood vessels in the body (Liu, Shao, Zhang, Jia & Dai, 2020; Ren, Wang, Hua, Xie & Tang, 2019); Vitamin D can be used to induce atherosclerotic lesions in animals (Bennani-Kabchi et al., 2000; Tian, Li & Zhang, 2021). It is important to note that currently available data from randomized trials in humans do not show any benefit of vitamin D supplementation for cardiovascular disease, and only some animal studies have shown a benefit or no benefit for cardiovascular disease.  Others: Increased vitamin D levels may play a role in the prevention of nonalcoholic fatty liver disease in European populations (Yuan & Larsson, 2022); Vitamin D supplements have a positive effect on patients with IBD and cystic fibrosis by regulating the gut microbiome and increasing the abundance of potentially beneficial bacterial strains, and further research on the infant microbiome and vitamin D/VDR will help reduce the risk of allergies and other diseases in adulthood (Sun, 2018). |
| 4 | Linolenic acid | Linolenic acid (LNA), an N-3 polyunsaturated fatty acid, is a precursor of DHA and EPA and plays an important role in maintaining human health. At present, the effects of linolenic acid on brain, eyes, cardiovascular system, normal growth and development of human body, regulation of blood lipids, blood sugar, anti-inflammatory, antioxidant and intestinal microflora have been widely reported. In addition, in recent years, linolenic acid has been studied as an alternative drug to prevent and treat microbial-related diseases. However, due to the lack of understanding of the antibacterial mechanism of linolenic acid, the application of linolenic acid as antibacterial agent has not been widely regarded (Chanda et al., 2018; Kim, Nam, Kim, Hayes & Lee, 2014; Tang, Jiang, Meng & Tao, 2018; Zorgetto-Pinheiro et al., 2022). |
| 5 | Linoleic acid | Linoleic acid (LA) belongs to N-6 polyunsaturated fatty acid. Currently, linoleic acid has been proven to have benefits such as anti-cancer, anti-oxidative stress, anti-atherosclerosis, anti-obesity, anti-diabetes and immune activity (Goncalves, Lira, Carnevali, Rosa, Pimentel & Seelaender, 2010); In addition, linoleic acid has neuroprotective activity (BVesga-Jimenez, MArtin, Barreto, Aristizabal-Pachon, Pinzon & Gonzalez, 2022); Linoleic acid reverses microglial inflammation induced by palmitic acid in a short-term high-fat diet (Tu, Kim, Yang, Kim & Kim, 2019). |
| 6 | Adenosine | Clinically, adenosine is suitable for the treatment of angina pectoris, hypertension, cerebrovascular disorders, apoplexy sequela, progressive muscular atrophy and so on.  Nervous system and cardiovascular system: adenosine dilate coronary arteries and reduces myocardial contractility (Tang et al., 2018); Adenosine prevents apoptosis induced by serum deprivation of rat PC12 cells by activating adenosine A receptor (Lu, Cheng, Lai, Lin & Huang, 2006); Adenosine has been widely used in the treatment of central nervous system and cardiovasular system diseases (Liu et al., 2019).  Immune system and neoplasm: Adenosine have immunomodulatory and anti-inflammatory activities. However, in some cases, the effects of adenosine on the immune system can be harmful because prolonged adenosine signaling can block anti-tumor and anti-bacterial immunity, thereby promoting the development and progression of cancer and sepsis, respectively (Antonioli, Fornai, Blandizzi, Pacher & Hasko, 2019).  Endocrine system: Adenosine regulates insulin secretion in beta cells of the pancreas (Szkudelski & Szkudelska, 2015).  Others: Adenosine can significantly inhibit platelet aggregation (Keunyoung et al., 2013); Adenosine is also promising for the treatment of acute and chronic pain (Adebiyi, Manalo, Kellems & Xia, 2019) and epilepsy (Weltha, Reemmer & Boison, 2019). |
| 7 | D-Phenylalanine | Nervous system: D-phenylalanine is an activator of carbonic anhydrase (CA), which is involved in various physiological processes, including learning and memory. Therefore, D-phenylalanine has a cognitive effect (Schmidt et al., 2020).  Neoplasm: D-phenylalanine may be a useful agent for the prevention of acute or unexpected pain in malignant diseases (Donzelle et al., 1981).  Endocrine and cardiovascular system: At present, D-phenylalanine is mainly used for the prevention and treatment of osteoporosis, cardiovascular diseases, diabetes, arteriosclerosis and other diseases. |
| 8 | Palmitic acid | Recent evidence suggests that palmitic acid is a signaling molecule that regulates the process and development of neurodegenerative diseases, metabolic syndrome, cardiovascular disease, cancer, inflammation, insulin resistance and other pathological diseases (Fatima et al., 2019; Titov, Krylin & Shiriaeva, 2011).  Nervous system: Excessive accumulation of exogenous palmitic acid produces lipid toxicity to brain tissue, involving toll-like receptor activation and activation of the NF-KB pathway (Vesga-Jimenez, Martin, Barreto, Aristizabal-Pachon, Pinzon & Gonzalez, 2022); In addition, palmitic acid induces apoptosis and oxidative stress in Schwann cells in diabetic peripheral neuropathy (Fan & Guo, 2022), hippocampal injury (Contreras et al., 2017), activates microglia and triggers an inflammatory response (Yanguas-Casas et al., 2018); Palmitic acid may be beneficial to enhance the differentiation of embryonic neural stem cells and their neurons (Ghareghani et al., 2017).  Immune system: Palmitic acid induces macrophage-like inflammatory responses by inducing Gn gene expression via TLR4 and NF-Kappa B (Akieda-Asai, Ma & Date, 2019); Palmitic acid can promote replication of Singapore grouper iris virus in fish cells in grouper spleen cells by inhibiting autophagy flux and tbK1-IRF3/7 pathway (Yu et al., 2020); Palmitic acid activates inflammasome NLRP3 and enhances the inflammatory response by increasing FABP4 / aP2 expression (Korbecki & Bajdak-Rusinek, 2019); Palmitic acid induces inflammation in the placental trophoblast cell line HTR8/SVneo and impinges its migration to smooth muscle cells via plasminogen activator inhibitor -I. In pathophysiological Settings such as obesity, high levels of palmitic acid may impair early placental development and predispositions to placental dysfunction (Rampersaud, Dunk, Lye & Renaud, 2021); Palmitic acid can activate the NF- Kappa B pathway by regulating TLR4 on human dendritic cells, and induce human dendritic cells to secrete pro-inflammatory factors such as IL-1β (Nicholas et al., 2017); Dietary palmitic acid can directly stimulate plasma cells to produce Ab and increase the number of IgA producing plasma cells in the intestine (Kunisawa et al., 2014); Toll-like receptor 2 and palmitic acid synergically activate inflammatory bodies in Kupffer cells and/or macrophages, thereby contributing to the development of non-alcoholic steatohepatitis (Miura, Yang, van Rooijen, Brenner, Ohnishi & Seki, 2013).  Endocrine system: Palmitic acid can increase TLR4 expression and promote resistin signal transduction through up-regulation of TLR4 and its recruitment in the membrane lipid raft, ultimately leading to insulin resistance and inflammation in sh-SY5Y human neuroblastoma (Amine, Benomar & Taouis, 2021). Palmitic acid can act with high glucose on human retinal glial cells and induce diabetic retinopathy related angiogenesis and the expression of PTGS2 and CXCL8 inflammatory targets (Capozzi, Giblin & Penn, 2018).  Neoplasm: Palmitic acid is a potential candidate for novel therapeutic agents that specifically attack multiple myeloma cells (Nagata et al., 2015); Ceramide and palmitic acid inhibit macrophage-mediated epithelial-mesenchymal transition in colorectal cancer (de Araujo et al., 2020); The antitumor effect of palmitic acid is related to its immunomodulatory activity (Boubaker et al., 2018).  Cardiovascular system: Dietary fats rich in palmitic acid can induce atherosclerosis by damaging macrophage cholesterol efflux and triggering inflammatory responses (Afonso et al., 2016); Palmitic acid can increase the inflammatory response of human aortic endothelial cells to lipopolysaccharide (Zhang, Franco, Schwartz, D'Souza, Karnick & Reaven, 2017).  Others: Brief exposure to palmitic acid induces intestinal dysfunction that targets barrier integrity and inflammation, and excessive palm oil consumption may be an early participant in the intestinal changes observed in metabolic diseases (Ghezzal et al., 2020). |
| 9 | Mannitol | Mannitol is commonly used as a hypertonic or potent diuretic to reduce intracranial hypertension or intraocular pressure, which can effectively reduce intracranial hypertension mortality, but can lead to systemic hypotension (Thongrong, Kong, Govindarajan, Allen, Mendel & Bergese, 2014).  Nervous system: Mannitol can improve cerebral ischemia/reperfusion injury in rats by stabilizing the blood-brain barrier and inflammatory response, and play a neuroprotective role (Shan et al., 2021); In addition, numerous studies have shown that mannitol can disrupt the blood-brain barrier to enhance the delivery of chemotherapeutic drugs to the brain. For example, mannitol breaks the blood-brain barrier to help stem cell therapy in the brain after chronic stroke (Tajiri, Lee, Acosta, Sanberg & Borlongan, 2016); Mannitol can enhance the therapeutic effect of intracarterial transplantation of mesenchymal stem cells into the brain after traumatic brain injury and improve motor function in rats (Okuma et al., 2013); Mannitol can be combined with arsenic trioxide in the treatment of acute promyelocytic leukemia relapse of central nervous system (Wang et al., 2014); Mannitol is used in the treatment of epilepsy (Ko & KAng, 2015); Mannitol reduces epileptic activity in the neocortex during early brain development through chloride and water co-transport (Glykys, Duquette, Duquette & Staley, 2018).  Immune system: Internal carotid hypertonic mannitol can open the blood-brain barrier, induce brain aseptic immunity and innate immune response, thus playing a potential neuro-immune regulatory role (Burks et al., 2021); Mannitol can trigger mast cell-dependent bronchoconstriction and prostacyclin bronchial protection in humans (Safholm et al., 2019); Mannitol can be used as a osmotic indirect bronchial activator to aid in the diagnosis and management of asthma, reflecting the underlying inflammatory process of asthma (Anderson, Daviskas, Brannan & Chan, 2018). Mannitol up-regulated HLA-DR in monocytes, CD11b in monocytes and neutrophils, and inhibited neutrophils apoptosis, suggesting that mannitol may interact directly with neutrophils and monocytes (Turina, Mulhall, Gardner, C. & N., 2008).  Neoplasm: Mannitol reduces cisplatin-induced acute kidney injury and AKI events observed in lung, upper gastrointestinal, and urinary tract cancers (Bégin et al., 2021; Hägerström, Lindberg, Bentzen, Brødbæk, Zerahn & Kristensen, 2019).  Cardiovascular system: Mannitol may cause an acute increase in blood pressure and pulse rate when used to lower intraocular pressure (Atik, Chan, Crock & Ang, 2020).  Others: Inhalation of mannitol increases airway mucosal ciliary clearance and mucus hydration. Mannitol has recently been proposed for the treatment of pulmonary cystic fibrosis (Nolan, Thornton, Murray & Dwyer, 2016) and osmotic hypotension, however, the long-term risks and benefits of mannitol require further research as the risks accumulate in the body (Nguyen, Veltchev & Nguyen, 2020); High dose infusion of mannitol has been associated with osmotic nephropathy and should be used with caution in patients with risk factors for kidney disease (Nomani et al., 2014). |
| 10 | Arecoline | Arecoline is the main component of areca alkaloid, which has hemostatic effect. However, it has been recently considered as a class II carcinogen, with cytotoxicity and genetic toxicity, and has damage to the immune system, liver, nerve and detoxification system of mice (Dasgupta et al., 2006; Li, Zang, Yin, Shen, Sun & Zhao, 2020).  Nervous System: Arecoline, a potentially addictive component of areca nuts, has Monoamine OXIDASE-A (Mao-a)-like properties that prevent the breakdown of neurotransmitters and increase dopamine and Serotonin levels in the brain (Ko, Lee, Ko & Ko, 2020); Arecoline has a significant anti-anxiety effect and can increase the levels of monoamine and microglia in brain (Serikuly et al., 2020); Arecoline can cause disruption of autophagy flux and is neurotoxic (Gao, Tang, Jiang, Zou, Zhang & Tang, 2022); Arecoline stimulates local inflammation and improves recovery from severe peripheral nerve injury (Lee, Tsai, Yao, Hsu, Chen & Wu, 2013).  Immune system: Arecoline has anti-inflammatory activity (Papke, Horenstein & Stokes, 2015), can affect the inflammatory cytokines produced by fibroblasts, act on immune cells Th17 and Treg, and change them (Wang, Gu & Tang, 2019).  Endocrine system: Arecoline can significantly promote the regeneration of β cells, and reverse the dysfunction of testis and other sexual organs by increasing the levels of insulin and gonadotropin in serum of type ⅰ diabetic rats (Saha, Das, Maiti & Chatterji, 2015).  Neoplasm: Arecoline-induced epigenetic changes play an important role in chemical-mediated cytotoxicity and genotoxic mechanisms and are important environmental risk factors for oral and hepatocellular carcinoma Taiwan (Kuo et al., 2019; Lin, Chang, Chen, Lee, Lin & Chang, 2011); Arecoline can induce epithelial mesenchymal transformation and promote oral cancer metastasis through serum amyloid A1 expression (Ren et al., 2021); Arecromine can induce the death of human leukemia K562 cells, which is related to the surface upregulation of TNFR2 (Chen & Chang, 2012).  Others: Arecoline significantly inhibited the ability of CB/ fMLP to trigger intracellular ROS production and myeloperoxidase release in human polymorphonuclear leukocytes (Lai, Lin, Yang, Liu & Hung, 2007); Arecoline attenuates or prevents bone loss by inhibiting osteoclast formation and promoting osteoblast formation (Liu, Chen, Lai, Lee & Chang, 2020); Arecoline is expected to reverse rheumatoid arthritis and osteoarthritis (Zuo, Zhu, Xiao, Wang, Shen & Chen, 2020). |
| 11 | Phthalic acid | Nervous system: None.  Immune system: None.  Endocrine system: None.  Neoplasm: None.  Cardiovascular system: None.  Others: Phthalic acid specifically produces significant in vitro and in vivo reproductive toxicity, especially sperm toxicity and testicular cell cytotoxicity (KWACK & LEE, 1998); Phthalic acid has an inhibitory effect on calcium signaling pathways coupled to nicotinic receptors (Liu, Tseng & Liu, 2009). |
| 12 | Cinnamic acid | Cinnamic acid can induce angiogenesis in vitro and has anti-cancer, hypoglycemic and anti-inflammatory activities.  Nervous system: Cinnamic acid protects dopaminergic neurons through PPARα and may be beneficial in the treatment of Parkinson's disease (Prorok, Jana, Patel & Pahan, 2019); Treatment with cinnamic acid significantly reduces the amyloid beta plaque burden and improved memory through PPARα in male familial Alzheimer's disease mice (Chandra, Roy, Jana & Pahan, 2019).  Immune system: Cinnamic acid pretreatment reduces bone marrow suppression and liver oxidative stress induced by cyclophosphamide (Patra et al., 2012).  Endocrine system: Cinnamic acid reduces glucose levels in diabetic rats in a time - and dose-dependent manner in vivo by improving glucose tolerance and in vitro by stimulating insulin secretion (Hafizur et al., 2015).  Neoplasm: Cinnamic acid has an effective anti-proliferation activity on melanoma cells (Niero & Machado-Santelli, 2013).  Cardiovascular system: Cinnamic acid has antiviral effects on viral myocarditis (VMC) induced by Coxsackie virus B-3 (CVB3) (Ding, Qiu, Zhao, Xu & Wang, 2010).  Others: Cinnamic acid reduces the activity of MDR-TB in a dose-dependent manner (Chen et al., 2011); Cinnamic acid has antibacterial activity against pathogenic bacteria, putrefying bacteria and Lactobacillus monocytogenes (Acero-Ortega, Dorantes-Alvarez, Hernández-Sánchez, Gutiérrez-López, Aparicio & Jaramillo-Flores, 2005; Dorantes et al., 2000; Narasimhan, Belsare, Pharande, Mourya & Dhake, 2004); Cinnamic acid has antifungal activities against beauveria bassiana, Lactobacillus sulphurum, Saccharomyces cerevisiae, Aspergillus flavus and Vetospora crude (Cheng, Liu, Chang & Chang, 2008; Kim, Campbell, Mahoney, Chan & Molyneux, 2004; Said, Neves & Griffiths, 2004). |
| 13 | Methyl linoleate | Nervous system: None.  Immune system: None.  Endocrine system: None.  Neoplasm: None.  Cardiovascular system: None.  Others: Anti-melanin production activity (Huh et al., 2010; Ko, Shrestha & Cho, 2018). |
| 14 | Palmitoleic acid | The effects of palmitoleic acid on health and disease are still controversial. Endogenous and dietary palmitoleic acid appear to have different metabolic effects (Hu, Fitzgerald, Topp, Alam & O'Hare, 2019). Circulating trans palmitoleic acid has been associated with lower insulin resistance, atherosclerotic dyslipidemia, and diabetes mellitus.  Nervous system: Elevated palmitoleic acid levels in patients with Alzheimer's disease, reduced palmitoleic acid levels and deoxygenation index in the brain may be associated with improved cognitive performance (Snigdha, Astarita, Piomelli & Cotman, 2012); BDNF can increase intracellular palmitoleic acid by activating palmitoleic acid synthesis and inhibiting palmitoleic acid extracellular release (Suzuki et al., 2012).  Immune system: Palmitoleic acid ameliorates palmitic acid-induced proinflammatory effects in non-obese rodent J774A-1 macrophages through TLR4-dependent and TNF-α independent signaling (Tsai, Lu, Chang, Hsu, Ho & Shih, 2021); P palmitoleic acid reduces inflammation in tissues with high metabolic rates; By activating AMP activated protein kinase (AMPK), palmioleic acid reverses giant cell polarization and gene expression of pro-inflammatory cytokines (Chan et al., 2015). MTORC1 regulates palmitoleic acid levels by controlling de novo synthesis, and inhibition of mTORC1 activity reduces palmitoleic acid levels. Palmitoleic acid in peripheral blood mononuclear cells (PBMC) is a potential biomarker of polymyositis (Yin, Wang, Cen, Yang, Yang & Xie, 2017).  Endocrine system: Palmitoleic acid is considered as a lipid hormone from fat, which regulates muscle insulin sensitivity and liver lipid metabolism (Cao, Gerhold, Mayers, Wiest, Watkins & Hotamisligil, 2008); Palmitoleic acid prevents palmitic acid-induced macrophage activation and P38 MAPK-mediated skeletal muscle insulin resistance (Talbot, Wheeler-Jones & Cleasby, 2014); Palmitoleic acid upregulates Bcl-2, protects human β cells from glucose and palmitic acid-induced apoptosis and restores the proliferation level of damaged β cells (Maedler, Oberholzer, Bucher, Spinas & Donath, 2003); Palmitoleic acid can inhibit hepatocyte lipid apoptosis and endoplasmic reticulum stress by inhibiting palmitic acid-induced upregulation of BCL-2 homolog 3 (BH-3) Bim protein and PUMA (Hu et al., 2019).  Neoplasm: Plasma monounsaturated fatty acids such as palmitoleic acid reflect the activity of the lipase stearoyl-coa-desaturase-1 (SCD-1). In recent years, palmitoleic acid has been proposed to be associated with cancer mortality and morbidity, but its specific role and molecular mechanism have been less reported (Byberg, Kilander, Warensjö Lemming, Michaëlsson & Vessby, 2014; Mamalakis, Kafatos, Kalogeropoulos, Andrikopoulos, Daskalopulos & Kranidis, 2002; Pouchieu et al., 2014; Waki et al., 2014).  Cardiovascular system: Recent epidemiological studies have shown that circulating palmitoleic acid is involved in cholesterol metabolism and homeostasis, but its effects on the cardiovascular system are unclear. A CHS cohort study showed that palmitoleic acid in plasma phospholipids decreased LDL and fibrinogen levels and increased HDL levels (Mozaffarian et al., 2010); Dietary or supplemental palmitoleic acid reduces plasma cholesterol and triglyceride levels (Curb and Wergowske et al., 2000). However, other studies have suggested that palmitoleic acid can lower HDL levels, increase blood pressure and increase triglyceridemia (Zong et al., 2012); The lipid factor palmitoleic acid is involved in mediating exercise-induced cardiac hypertrophy by inducing phosphorylation activation of the serine/threonine protein kinase Akt in cardiomyocytes (Foryst-Ludwig et al., 2015); Palmitoleic acid can mediate endothelial function as a gap junction inhibitor (Kenny, Baker, Kendall, Randall & Dunn, 2002).  Others: Palmitoleic acid is an adipose tissue-derived monounsaturated free fatty acid that plays a role as a lipid factor in metabolic and inflammatory diseases. Serum palmitoleic acid can be used as a marker of local inflammation and prognosis of Crohn's disease in inflammatory bowel disease (Akazawa et al., 2021); Palmitoleic acid can inhibit melanin production (Yoon et al., 2010). |

**References:**

Acero-Ortega, C., Dorantes-Alvarez, L., Hernández-Sánchez, H., Gutiérrez-López, G., Aparicio, G., & Jaramillo-Flores, M. E. (2005). Evaluation of Phenylpropanoids in Ten Capsicum annuum L. Varieties and Their Inhibitory Effects on Listeria monocytogenes Murray, Webb and Swann Scott A. *FOOD SCIENCE AND TECHNOLOGY INTERNATIONAL*, *11*(1), 5-10.

Adachi, Y., Sasagawa, I., & Nakada, T. (1993). Reproductive insufficiency in the male rat with adenine-induced chronic renal failure. *UROLOGIA INTERNATIONALIS*, *51*(4), 228-230.

Adebiyi, M. G., Manalo, J., Kellems, R. E., & Xia, Y. (2019). Differential role of adenosine signaling cascade in acute and chronic pain. *NEUROSCIENCE LETTERS*, *712*.

Afonso, M. S., Lavrador, M. S. F., Koike, M. K., Cintra, D. E., Ferreira, F. D., Nunes, V. S., Castilho, G., Gioielli, L. A., Bombo, R. P., Catanozi, S., Caldini, E. G., Damaceno-Rodrigues, N. R., Passarelli, M., Nakandakare, E. R., & Lottenberg, A. M. (2016). Dietary interesterified fat enriched with palmitic acid induces atherosclerosis by impairing macrophage cholesterol efflux and eliciting inflammation. *JOURNAL OF NUTRITIONAL BIOCHEMISTRY*, *36*, 89-90.

Akazawa, Y., Morisaki, T., Fukuda, H., Norimatsu, K., Shiota, J., Hashiguchi, K., Tabuchi, M., Kitayama, M., Matsushima, K., Yamaguchi, N., Kondo, H., Fujita, F., Takeshita, H., Nakao, K., & Takeshima, F. (2021). Significance of serum palmitoleic acid levels in inflammatory bowel disease. *Scientific Reports*, *11*(1).

Akieda-Asai, S., Ma, H., & Date, Y. (2019). Palmitic acid induces guanylin gene expression through the Toll-like receptor 4 nuclear factor-kappa B pathway in rat macrophages. *AMERICAN JOURNAL OF PHYSIOLOGY-CELL PHYSIOLOGY*, *317*(6), C1239-C1246.

Akintonwa, A., & Auditore, J. V. (1978). Reversal of adenine-induced depression of mouse locomotor activity by amphetamine. *Arch Int Pharmacodyn Ther*, *235*(2), 248-253.

Amine, H., Benomar, Y., & Taouis, M. (2021). Palmitic acid promotes resistin-induced insulin resistance and inflammation in SH-SY5Y human neuroblastoma. *Scientific Reports*, *11*(1).

Anderson, S. D., Daviskas, E., Brannan, J. D., & Chan, H. K. (2018). Repurposing excipients as active inhalation agents the mannitol story. *ADVANCED DRUG DELIVERY REVIEWS*, *133*, 45-56.

Antonioli, L., Fornai, M., Blandizzi, C., Pacher, P., & Hasko, G. (2019). Adenosine signaling and the immune system When a lot could be too much. *IMMUNOLOGY LETTERS*, *205*, 9-15.

Atik, A., Chan, E., Crock, C., & Ang, G. S. (2020). Cardiovascular effects and safety of mannitol in treating raised intraocular pressure. *Clinical & Experimental Ophthalmology*, *48*(3), 409-411.

Bégin, A. M., Monfette, M. L., Boudrias-Dalle, É., Lavallée, E., Samouelian, V., Soulières, D., Chagnon, M., Fournier, M. A., Letarte, N., & Adam, J. P. (2021). Effect of mannitol on acute kidney injury induced by cisplatin. *SUPPORTIVE CARE IN CANCER*, *29*, 2083-2091.

Bennani-Kabchi, N., Kehel, L., El Bouayadi, F., Fdhil, H., Amarti, A., Saidi, A., & Marquie, G. (2000). New model of atherosclerosis in insulin resistant sand rats’ hypercholesterolemia combined with D2 vitamin. *ATHEROSCLEROSIS*, *150*(1), 55-61.

Boubaker, J., Ben, T. I., Sassi, A., Bzouich-Mokded, I., Mazgar, S. G., Sioud, F., Bedoui, A., Skhiri, S. S., Ghedira, K., & Chekir-Ghedira, L. (2018). Antitumoral Potency by Immunomodulation of Chloroform Extract from Leaves of Nitraria retusa, Tunisian Medicinal Plant, via its Major Compounds -sitosterol and Palmitic Acid in BALB/c Mice Bearing Induced Tumor. *NUTRITION AND CANCER-AN INTERNATIONAL JOURNAL*, *70*(4), 650-662.

Burks, S. R., Kersch, C. N., Witko, J. A., Pagel, M. A., Sundby, M., Muldoon, L. L., Neuwelt, E. A., & Frank, J. A. (2021). Blood-brain barrier opening by intracarotid artery hyperosmolar mannitol induces sterile inflammatory and innate immune responses. *Immunology and Inflammation*, *118*(18), e2021915118.

BVesga-Jimenez, D. J., MArtin, C., Barreto, G. E., Aristizabal-Pachon, A. F., Pinzon, A., & Gonzalez, J. (2022). Fatty Acids An Insight into the Pathogenesis of Neurodegenerative Diseases and Therapeutic Potential. *INTERNATIONAL JOURNAL OF MOLECULAR SCIENCES*, *23*(5).

Byberg, L., Kilander, L., Warensjö Lemming, E., Michaëlsson, K., & Vessby, B. (2014). Cancer death is related to high palmitoleic acid in serum and to polymorphisms in the SCD-1 gene in healthy Swedish men. *The American Journal of Clinical Nutrition*, *99*(3), 551-558.

Caccamo, D., Ricca, S., Currò, M., & Ientile, R. (2018). Health Risks of Hypovitaminosis D: A Review of New Molecular Insights. *INTERNATIONAL JOURNAL OF MOLECULAR SCIENCES*, *19*(3), 892.

Cao, H., Gerhold, K., Mayers, J. R., Wiest, M. M., Watkins, S. M., & Hotamisligil, G. S. (2008). Identification of a lipokine, a lipid hormone linking adipose tissue to systemic metabolism. *CELL*, *134*(6), 933-944.

Capozzi, M. E., Giblin, M. J., & Penn, J. S. (2018). Palmitic Acid Induces Muller Cell Inflammation that is Potentiated by Co-treatment with Glucose. *Scientific Reports*, *8*.

Chalmers, A. H., Rotstein, T., Mohan, R. M., Marshall, V. R., & Coleman, M. (1985). Studies on the mechanism of immunosuppression with adenine. *International journal of immunopharmacology*, *7*(4), 433-442.

Chan, K. L., Pillon, N. J., Sivaloganathan, D. M., Costford, S. R., Liu, Z., Théret, M., Chazaud, B., & Klip, A. (2015). Palmitoleate Reverses High Fat-induced Proinflammatory Macrophage Polarization via AMP-activated Protein Kinase (AMPK). *JOURNAL OF BIOLOGICAL CHEMISTRY*, *290*(27), 16979-16988.

Chanda, W., Joseph, T. P., Guo, X. F., Wang, W. D., Liu, M., Vuai, M. S., Padhiar, A. A., & Zhong, M. T. (2018). Effectiveness of omega-3 polyunsaturated fatty acids against microbial pathogens. *Journal of Zhejiang University-SCIENCE B*, *19*(4), 253-262.

Chandra, S., Roy, A., Jana, M., & Pahan, K. (2019). Cinnamic acid activates PPARα to stimulate Lysosomal biogenesis and lower Amyloid plaque pathology in an Alzheimer's disease mouse model. *NEUROBIOLOGY OF DISEASE*, *124*, 379-395.

Charoenngam, N., Shirvani, A., & Holick, M. F. (2019). Vitamin D for skeletal and non-skeletal health What we should know. *Journal of clinical orthopaedics and trauma*, *10*(6), 1082-1093.

Chen, S. Y., Lin, C. H., Lin, J. T., Cheng, Y. F., Chen, H. M., & Kao, S. H. (2017). Adenine causes cell cycle arrest and autophagy of chronic myelogenous leukemia K562 cells via AMP-activated protein kinase signaling. *Oncology Letters*, *14*(5), 5575-5580.

Chen, Y. J., & Chang, L. S. (2012). Arecoline-induced death of human leukemia K562 cells is associated with surface up-modulation of TNFR2. *JOURNAL OF CELLULAR PHYSIOLOGY*, *227*(5), 2240-2251.

Chen, Y. P., Chu, Y. L., Tsuang, Y. H., Wu, Y., Kuo, C. Y., & Kuo, Y. J. (2020). Anti-Inflammatory Effects of Adenine Enhance Osteogenesis in the Osteoblast-Like MG-63 Cells. *LIFE-BASEL*, *10*(7).

Chen, Y., Huang, S., Sun, F., Chiang, Y., Chiang, C., Tsai, C., & Weng, C. (2011). Transformation of cinnamic acid from trans- to cis-form raises a notable bactericidal and synergistic activity against multiple-drug resistant Mycobacterium tuberculosis. *EUROPEAN JOURNAL OF PHARMACEUTICAL SCIENCES*, *43*(3), 188-194.

Cheng, S., Liu, J., Chang, E., & Chang, S. (2008). Antifungal activity of cinnamaldehyde and eugenol congeners against wood-rot fungi. *BIORESOURCE TECHNOLOGY*, *99*(11), 5145-5149.

Cheng, Y. F., Young, G. H., Lin, J. T., Jang, H. H., Chen, C. C., Nong, J. Y., Chen, P. K., Kuo, C. Y., Kao, S. H., Liang, Y. J., & Chen, H. M. (2015). Activation of AMP-Activated Protein Kinase by Adenine Alleviates TNF-Alpha-Induced Inflammation in Human Umbilical Vein Endothelial Cells. *PLoS One*, *10*(11).

Contreras, A., Del Rio, D., Martinez, A., Gil, C., Morales, L., Ruiz-Gayo, M., & Del, O. N. (2017). Inhibition of hippocampal long-term potentiation by high-fat diets is it related to an effect of palmitic acid involving glycogen synthase kinase-3. *NEUROREPORT*, *28*(6), 354-359.

Dasgupta, R., Saha, I., Pal, S., Bhattacharyya, A., Sa, G., Nag, T., Das, T., & Maiti, B. V. (2006). Immunosuppression, hepatotoxicity and depression of antioxidant status by arecoline in albino mice. *TOXICOLOGY*, *227*(1-2), 94-104.

de Araujo, R. F., Eich, C., Jorquera, C., Schomann, T., Baldazzi, F., Chan, A. B., & Cruz, L. J. (2020). Ceramide and palmitic acid inhibit macrophage-mediated epithelial-mesenchymal transition in colorectal cancer. *MOLECULAR AND CELLULAR BIOCHEMISTRY*, *469*(1-2), 179-180.

Ding, Y., Qiu, L., Zhao, G., Xu, J., & Wang, S. (2010). Influence of cinnamaldehyde on viral myocarditis in mice. *AMERICAN JOURNAL OF THE MEDICAL SCIENCES*, *340*(2), 114-120.

Donzelle, G., Bernard, L., Deumier, R., Lacome, M., Barre, M., Lanier, M., & Mourtada, M. B. (1981). Curing trial of complicated oncologic pain by D-phenylalanine. *Anesthesie, analgesie, reanimation*, *38*(11-12), 655-658.

Dorantes, L., Colmenero, R., Hernandez, H., Mota, L., Jaramillo, M. E., Fernandez, E., & Solano, C. (2000). Inhibition of growth of some foodborne pathogenic bacteria by Capsicum annum extracts. *INTERNATIONAL JOURNAL OF FOOD MICROBIOLOGY*, *57*(1), 125-128.

Drori, A., Shabat, Y., Ben Ya'Acov, A., Danny, O., Levanon, D., Zolotarov, L., & Ilan, Y. (2016). Extracts from Lentinula edodes (Shiitake) Edible Mushrooms Enriched with Vitamin D Exert an Anti-Inflammatory Hepatoprotective Effect. *JOURNAL OF MEDICINAL FOOD*, *19*(4), 383-389.

EI Agaty, S. M. (2019). Cardioprotective effect of vitamin D-2 on isoproterenol-induced myocardial infarction in diabetic rats. *ARCHIVES OF PHYSIOLOGY AND BIOCHEMISTRY*, *125*(3), 210-219.

Eliza, J., Daisy, P., Ignacimuthu, S., & Duraipandiyan, V. (2009). Normo-glycemic and hypolipidemic effect of costunolide isolated from Costus speciosus (Koen ex. Retz.) Sm. in streptozotocin-induced diabetic rats. *CHEMICO-BIOLOGICAL INTERACTIONS*, *179*(2-3), 329-334.

Fan, J. W., & Guo, L. X. (2022). Palmitic Acid-Induced SchwannInduced Cells Apoptosis and Oxidative Stress in Diabetes Peripheral Neuropathy. *DIABETES*, *70*, 416.

Fatima, S., J., H. X., Gong, R. H., Huang, C. H., Chen, M. T., Wong, H. L. X., Bian, Z. X., & Kwan, H. Y. (2019). Palmitic acid is an intracellular signaling molecule involved in disease development. *CELLULAR AND MOLECULAR LIFE SCIENCES*, *76*(13), 2547-2557.

Foryst-Ludwig, A., Kreissl, M. C., Benz, V., Brix, S., Smeir, E., Ban, Z., Januszewicz, E., Salatzki, J., Grune, J., Schwanstecher, A., Blumrich, A., Schirbel, A., Klopfleisch, R., Rothe, M., Blume, K., Halle, M., Wolfarth, B., Kershaw, E. E., & Kintscher, U. (2015). Adipose Tissue Lipolysis Promotes Exercise-induced Cardiac Hypertrophy Involving the Lipokine C16:1n7-Palmitoleate. *JOURNAL OF BIOLOGICAL CHEMISTRY*, *290*(39), 23603-23615.

Fukuda, T., Kuroda, T., Kono, M., Hyoguchi, M., Tajiri, S., Tanaka, M., Mine, Y., & Matsui, T. (2016). Adenine attenuates the Ca2+ contraction-signaling pathway via adenine receptor-mediated signaling in rat vascular smooth muscle cells. *NAUNYN-SCHMIEDEBERGS ARCHIVES OF PHARMACOLOGY*, *389*(9), 999-1007.

Fukuda, T., Majumder, K., Zhang, H., Turner, P. V., Matsui, T., & Mine, Y. (2016). Adenine Inhibits TNF-alpha Signaling in Intestinal Epithelial Cells and Reduces Mucosal Inflammation in a Dextran Sodium Sulfate-Induced Colitis Mouse Model. *JOURNAL OF AGRICULTURAL AND FOOD CHEMISTRY*, *64*(21), 4227-4234.

Gao, S. L., Tang, Y. Y., Jiang, J. M., Zou, W., Zhang, P., & Tang, X. Q. (2022). Improvement of autophagic flux mediates the protection of hydrogen sulfide against arecoline-elicited neurotoxicity in PC12 cells. *CELL CYCLE*, *21*(10), 1077-1090.

Ghareghani, M., Zibara, K., Azari, H., Hejr, H., Sadri, F., Jannesar, R., Ghalamfarsa, G., Delaviz, H., Nouri, E., & Ghanbari, A. (2017). Safflower Seed Oil,Containing Oleic Acid and Palmitic Acid, Enhances the Stemness of Cultured Embryonic Neural Stem Cells through Notch1 and Induces Neuronal Differentiation. *Frontiers in Neuroscience*, *11*.

Ghezzal, S., Postal, B. G., Quevrain, E., Brot, L., Seksik, P., Letuique, A., Thenet, S., & Carriere, V. (2020). Palmitic acid damages gut epithelium integrity and initiates inflammatory cytokine production. *BIOCHIMICA ET BIOPHYSICA ACTA-MOLECULAR AND CELL BIOLOGY OF LIPIDS*, *1865*(2).

Glykys, J., Duquette, E., Duquette, K., & Staley, K. (2018). Mannitol Decreases Neocortical Epileptiform Activity during Early Brain Development via Cotransport of Chloride and Water. *ANNALS OF NEUROLOGY*, *84*, S268.

Goncalves, D. C., Lira, F. S., Carnevali, L. C., Rosa, J. C., Pimentel, G. D., & Seelaender, M. (2010). Conjugated Linoleic Acid: good or bad nutrient. *Diabetology & Metabolic Syndrome*, *2*.

Hafizur, R. M., Hameed, A., Shukrana, M., Raza, S. A., Chishti, S., Kabir, N., & Siddiqui, R. A. (2015). Cinnamic acid exerts anti-diabetic activity by improving glucose tolerance in vivo and by stimulating insulin secretion in vitro. *PHYTOMEDICINE*, *22*(2), 297-300.

Hägerström, E., Lindberg, L., Bentzen, J., Brødbæk, K., Zerahn, B., & Kristensen, B. (2019). The Nephroprotective Effect of Mannitol in Head and Neck Cancer Patients Receiving Cisplatin Therapy. *CLINICAL MEDICINE INSIGHTS-ONCOLOGY*, *13*.

Hartmann, J., & Getoff, N. (2006). Radiation-induced effect of adenine (vitamin B-4) on mitomycin C activity. In vitro experiments. *ANTICANCER RESEARCH*, *26*(4B), 3005-3010.

Hirasawa, K., Yoshida, O., Fujinami, T., Sohma, K., & Watanabe, A. (2000). Adenine-induced selective apoptosis toward HIV chronically infected cells in vitro. *BIOCHEMICAL AND BIOPHYSICAL RESEARCH COMMUNICATIONS*, *273*(3), 1025-1032.

Hu, M., Liu, L. S., & Yao, W. R. (2018). Activation of p53 by costunolide blocks glutaminolysis and inhibits proliferation in human colorectal cancer cells. *GENE*, *678*, 261-269.

Hu, W., Fitzgerald, M., Topp, B., Alam, M., & O'Hare, T. J. (2019). A review of biological functions, health benefits, and possible de novo biosynthetic pathway of palmitoleic acid in macadamia nuts. *Journal of Functional Foods*, *62*, 103520.

Huang, C. W., Lin, Y. C., Hung, C. H., Chen, H. M., Lin, J. T., Wang, C. J., & Kao, S. H. (2021). Adenine Inhibits the Invasive Potential of DLD-1 Human Colorectal Cancer Cell via the AMPK FAK Axis. *PHARMACEUTICALS*, *14*(9).

Huh, S., Kim, Y., Jung, E., Lim, J., Jung, K. S., Kim, M., Lee, J., Park, D., CDepartment, O. C. B., Sookmyung, W. U., BSkinCure, L. S. I., Jeju, B. D. C., & ABiospectrum, L. S. I. (2010). Melanogenesis Inhibitory Effect of Fatty Acid Alkyl Esters Isolated from Oxalis triangularis. *BIOLOGICAL & PHARMACEUTICAL BULLETIN*, *33*(7), 1242-1245.

Jeong, S., Itokawa, T., Shibuya, M., Kuwano, M., Ono, M., Higuchi, R., & Miyamoto, T. (2002). Costunolide, a sesquiterpene lactone from Saussurea lappa, inhibits the VEGFR KDR Flk-1 signaling pathway. *CANCER LETTERS*, *187*(1-2), 129-133.

Kaldor, A., Rihan, Z. E., Nichols, T. R., & Butterfield, W. J. (1964). EFFECTS OF ADENINE AND GUANINE ON HEPATIC GLUCOSE RELEASE AND ON THE ACTION OF INSULIN ON THE LIVER. *NATURE*, *203*, 1186.

Kenny, L. C., Baker, P. N., Kendall, D. A., Randall, M. D., & Dunn, W. R. (2002). The role of gap junctions in mediating endothelium-dependent responses to bradykinin in myometrial small arteries isolated from pregnant women. *Br J Pharmacol*, *136*(8), 1085-1088.

Keunyoung, K., Kyung-Min, L., Hyun-Jung, S., Dae-Bang, S., Ji-Yoon, N., Seojin, K., Han, Y. C., Sue, S., Jin-Ho, C., & Ok-Nam, B. (2013). Inhibitory effects of black soybean on platelet activation mediated through its active component of adenosine. *THROMBOSIS RESEARCH*, *131*(3), 254-261.

Kim, J. H., Campbell, B. C., Mahoney, N. E., Chan, K. L., & Molyneux, R. J. (2004). Identification of Phenolics for Control ofAspergillus flavus UsingSaccharomyces cerevisiae in a Model Target-Gene Bioassay. *JOURNAL OF AGRICULTURAL AND FOOD CHEMISTRY*, *52*(26), 7814-7821.

Kim, K. B., Nam, Y. A., Kim, H. S., Hayes, A. W., & Lee, B. M. (2014). alpha-Linolenic acid Nutraceutical, pharmacological and toxicological evaluation. *FOOD AND CHEMICAL TOXICOLOGY*, *70*, 163-178.

Ko, A. M. S., Lee, C. H., Ko, A. M. J., & Ko, Y. C. (2020). Betel quid dependence mechanism and potential cessation therapy. *PROGRESS IN NEURO-PSYCHOPHARMACOLOGY & BIOLOGICAL PSYCHIATRY*, *103*.

Ko, A. R., & KAng, T. C. (2015). Mannitol induces selective astroglial death in the CA1 region of the rat hippocampus following status epilepticus. *BMB Reports*, *48*(9), 507-512.

Ko, G. A., Shrestha, S., & Cho, S. K. (2018). Sageretia thea fruit extracts rich in methyl linoleate and methyl linolenate downregulate melanogenesis via the Akt/GSK3 β signaling pathway. *Anti-melanogenesis effect of Sageretia thea*, *12*(1), 3-12.

Korbecki, J., & Bajdak-Rusinek, K. (2019). The effect of palmitic acid on inflammatory response in macrophages an overview of molecular mechanisms. *INFLAMMATION RESEARCH*, *68*(11), 915-932.

Kunisawa, J., Hashimoto, E., Inoue, A., Nagasawa, R., Suzuki, Y., Ishikawa, I., Shikata, S., Arita, M., Aoki, J., & Kiyono, H. (2014). Regulation of Intestinal IgA Responses by Dietary Palmitic Acid and Its Metabolism. *JOURNAL OF IMMUNOLOGY*, *193*(4), 1666-1671.

Kuo, T., Nithiyanantham, S., Lee, C., Hsu, H., Luo, S., Lin, Y., Yeh, K., & Ko, Y. (2019). Arecoline N-oxide regulates oral squamous cell carcinoma development through NOTCH1 and FAT1 expressions. *JOURNAL OF CELLULAR PHYSIOLOGY*, *234*(8), 13984-13993.

KWACK, S. J., & LEE, B. M. (1998). Comparative Cytotoxicity and Sperm Motility Using a Computer-Aided Sperm Analysis System (CASA) for Isomers of Phthalic Acid, a Common Final Metabolite of Phthalates. *Journal of toxicology and environmental health*, *001*, 1-3.

Lai, H. W., Wei, J. C. C., Hung, H. C., & Lin, C. C. (2019). Adenine Inhibits the Growth of Colon Cancer Cells via AMP-Activated Protein Kinase Mediated Autophagy., *2019*.

Lai, Y., Lin, J., Yang, S., Liu, T., & Hung, S. (2007). Areca nut extracts reduce the intracellular reactive oxygen species and release of myeloperoxidase by human polymorphonuclear leukocytes. *JOURNAL OF PERIODONTAL RESEARCH*, *42*(1), 69-76.

Lee, S. H., Cho, Y. C., & Lim, J. S. (2021). Costunolide, a Sesquiterpene Lactone, Suppresses Skin Cancer via Induction of Apoptosis and Blockage of Cell Proliferation. *INTERNATIONAL JOURNAL OF MOLECULAR SCIENCES*, *22*(4).

Lee, S., Tsai, C., Yao, C., Hsu, Y., Chen, Y., & Wu, M. (2013). Effect of Arecoline on Regeneration of Injured Peripheral Nerves. *AMERICAN JOURNAL OF CHINESE MEDICINE*, *41*(4), 865-885.

Leu, J. G., Wang, C. M., Chen, C. Y., Yang, Y. F., Shih, C. Y., Lin, J. T., Chen, H. M., & Liang, Y. J. (2022). The Cell Protective Effect of Adenine on Hypoxia-Reoxygenation Injury through PPAR Delta Activation. *LIFE-BASEL*, *11*(12).

Li, C. J., Wang, B., & Fan, B. Q. (1994). The meiosis arrest of mouse oocytes induced by adenine., *27*(4), 457-462.

Li, W. D., Zang, C. J., Yin, S., Shen, W., Sun, Q. Y., & Zhao, M. (2020). Metformin protects against mouse oocyte apoptosis defects induced by arecoline. *CELL PROLIFERATION*, *53*(7).

Li, X., Liu, Q. Q., Yu, J. Y., Zhang, R. T., Sun, T., Jiang, W., Hu, N., Yang, P., Luo, L., & Ren, J. (2021). Costunolide ameliorates intestinal dysfunction and depressive behaviour in mice with stress-induced irritable bowel syndrome via c. *Food & Function*, *12*(9), 4142-4151.

Lin, P. C., Chang, W. H., Chen, Y. H., Lee, C. C., Lin, Y. H., & Chang, J. G. (2011). Cytotoxic Effects Produced by Arecoline Correlated to Epigenetic Regulation in Human K-562 Cells. *JOURNAL OF TOXICOLOGY AND ENVIRONMENTAL HEALTH-PART A-CURRENT ISSUES*, *74*(11), 737-745.

Liu, B., Rong, Y. M., Sun, D., Li, W. W., Chen, H., Cao, B., & Wang, T. Y. (2019). Costunolide inhibits pulmonary fibrosis via regulating NF-kB and TGF-beta (1)/Smad (2)/Nrf (2)-NOX4 signaling pathways. *BIOCHEMICAL AND BIOPHYSICAL RESEARCH COMMUNICATIONS*, *510*(2), 329-333.

Liu, F., Chen, C., Lai, C., Lee, C., & Chang, D. (2020). Arecoline suppresses RANKL-induced osteoclast differentiation in vitro and attenuates LPS-induced bone loss in vivo. *PHYTOMEDICINE*, *69*.

Liu, P. S., Tseng, F. W., & Liu, J. H. (2009). Comparative suppression of phthalate monoesters and phthalate diesters on calcium signalling coupled to nicotinic acetylcholine receptors. *JOURNAL OF TOXICOLOGICAL SCIENCES*, *34*(3), 255-263.

Liu, Y. J., Chen, J., Li, X., Zhou, X., Hu, Y. M., Chu, S. F., Peng, Y., & Chen, N. H. (2019). Research progress on adenosine in central nervous system diseases. *CNS Neuroscience & Therapeutics*, *25*(9), 899-910.

Liu, Y. R., Shao, Q., Zhang, H. H., Jia, Y., & Dai, M. (2020). Inhibitory effect of paeonol on aortic endothelial inflammation in atherosclerotic rats by up-regulation of caveolin-1 expression and suppression of NF-kappaB pathway. *China journal of Chinese materia medica*, *45*(11), 2578-2585.

Lu, M. K., Cheng, J. J., Lai, W. L., Lin, Y. R., & Huang, N. K. (2006). Adenosine as an active component of Antrodia cinnamomea that prevents rat PC12 cells from serum deprivation-induced apoptosis through the activation of adenosine A receptors2A. *LIFE SCIENCES*, *79*(3), 252-258.

Lv, Q., Xing, Y., Dong, D., Hu, Y., Chen, Q. Z., Zhai, L. H., Hu, L. H., & Zhang, Y. A. (2021). Costunolide ameliorates colitis via specific inhibition of HIF1 alpha glycolysis-mediated Th17 differentiation. *INTERNATIONAL IMMUNOPHARMACOLOGY*, *97*.

Maedler, K., Oberholzer, J., Bucher, P., Spinas, G. A., & Donath, M. Y. (2003). Monounsaturated fatty acids prevent the deleterious effects of palmitate and high glucose on human pancreatic beta-cell turnover and function. *DIABETES*, *52*(3), 726-733.

Mamalakis, G., Kafatos, A., Kalogeropoulos, N., Andrikopoulos, N., Daskalopulos, G., & Kranidis, A. (2002). Prostate cancer vs hyperplasia: relationships with prostatic and adipose tissue fatty acid composition. *Prostaglandins, leukotrienes, and essential fatty acids*, *66*(5-6), 467-477.

Meng, J. X., Zhan, H. H., Meng, F. C., Wang, G. W., Huang, D., Liao, Z. H., & CHen, M. (2022). Costunolide protects against alcohol-induced liver injury by regulating gut microbiota, oxidative stress and attenuating inflammation in vivo and in vitro. *PHYTOTHERAPY RESEARCH*, *36*(3), 1268-1283.

Meng, L. Q., Ma, H. X., Meng, J. N., Li, T., Zhu, Y. F., & Zhao, Q. P. (2021). Costunolide attenuates oxygen-glucose deprivationreperfusion-induced mitochondrial-mediated apoptosis in PC12 cells. *Molecular Medicine Reports*, *23*(6).

Miura, K., Yang, L., van Rooijen, N., Brenner, D. A., Ohnishi, H., & Seki, E. (2013). Toll-Like Receptor 2 and Palmitic Acid Cooperatively Contribute to the Development of Nonalcoholic Steatohepatitis Through Inflammasome Activation in Mice. *HEPATOLOGY*, *57*(2), 577-589.

Mohammad, S., Mishra, A., & Ashraf, M. Z. (2019). Emerging Role of Vitamin D and its Associated Molecules in Pathways Related to Pathogenesis of Thrombosis. *Biomolecules (Basel, Switzerland)*, *9*(11), 649.

Mozaffarian, D., Cao, H., King, I. B., Lemaitre, R. N., Song, X., Siscovick, D. S., & Hotamisligil, G. S. (2010). Circulating palmitoleic acid and risk of metabolic abnormalities and new-onset diabetes. *The American Journal of Clinical Nutrition*, *92*(6), 1350-1358.

Muller, D. C., Hodge, A. M., Fanidi, A., Albanes, D., Mai, X. M., Shu, X. O., Weinstein, S. J., Larose, T. L., Zhang, X., Han, J., Stampfer, M. J., Smith-Warner, S. A., Ma, J., Gaziano, J. M., Sesso, H. D., Stevens, V. L., McCullough, M. L., Layne, T. M., Prentice, R., Pettinger, M., Thomson, C. A., Zheng, W., Gao, Y. T., Rothman, N., Xiang, Y. B., Cai, H., Wang, R., Yuan, J. M., Koh, W. P., Butler, L. M., Cai, Q., Blot, W. J., Wu, J., Ueland, P. M., Midttun, Ø., Langhammer, A., Hveem, K., Johansson, M., Hultdin, J., Grankvist, K., Arslan, A. A., Le Marchand, L., Severi, G., Johansson, M., & Brennan, P. (2018). No association between circulating concentrations of vitamin D and risk of lung cancer: an analysis in 20 prospective studies in the Lung Cancer Cohort Consortium (LC3). *ANNALS OF ONCOLOGY*, *29*(6), 1468-1475.

Nagata, Y., Ishizaki, I., Waki, M., Ide, Y., Hossen, M. A., Ohnishi, K., Muyayama, T., & Setou, M. (2015). Palmitic acid, verified by lipid profiling using secondary ion mass spectrometry, demonstrates anti-multiple myeloma activity. *LEUKEMIA RESEARCH*, *39*(6), 638-645.

Narasimhan, B., Belsare, D., Pharande, D., Mourya, V., & Dhake, A. (2004). Esters, amides and substituted derivatives of cinnamic acid: synthesis, antimicrobial activity and QSAR investigations. *EUROPEAN JOURNAL OF MEDICINAL CHEMISTRY*, *39*(10), 827-834.

Nemmar, A., Karaca, T., Beegam, S., Yuvaraju, P., Yasin, J., Hamadi, N. K., & Ali, B. H. (2016). Prolonged Pulmonary Exposure to Diesel Exhaust Particles Exacerbates Renal Oxidative Stress, Inflammation and DNA Damage in Mice with Adenine-Induced Chronic Renal Failure. *CELLULAR PHYSIOLOGY AND BIOCHEMISTRY*, *38*(5), 1703-1713.

Nguyen, T., Veltchev, K., & Nguyen, T. V. (2020). Mannitol Role in the Management of Intradialytic Hypotension. *AMERICAN JOURNAL OF THERAPEUTICS*, *27*(5), E450-E454.

Nicholas, D. A., Zhang, K., Hung, C., Glasgow, S., Aruni, A. W., Unternaehrer, J., Payne, K. J., Langridge, W. H. R., & De, L. M. (2017). Palmitic acid is a toll-like receptor 4 ligand that induces human dendritic cell secretion of IL-1 beta. *PLoS One*, *12*(5).

Niero, E. L., & Machado-Santelli, G. M. (2013). Cinnamic acid induces apoptotic cell death and cytoskeleton disruption in human melanoma cells. *J Exp Clin Cancer Res*, *32*, 31.

Nolan, S. J., Thornton, J., Murray, C. S., & Dwyer, T. (2016). Inhaled Mannitol (Bronchitol) for Cystic Fibrosis. *Paediatric Respiratory Reviews*, *18*, 52-54.

Nomani, A. Z., Nabi, Z., Rashib, H., Janjua, J., Nomani, H., Majeed, A., Chaudry, S. R., & Mazhar, A. S. (2014). Osmotic nephrosis with mannitol review article. *RENAL FAILURE*, *36*(7), 1169-1176.

Ohyama, H., & Yamada, T. (1970). The restorative effect of adenine on radiation damage in rat thymocytes. *International journal of radiation biology and related studies in physics, chemistry, and medicine*, *17*(3), 277-278.

Okuma, Y., Wang, F. F., Toyoshima, A., Kameda, M., Hishikawa, T., Tokunaga, K., Sugiu, K., Liu, K. Y., Haruma, J., Nishibori, M., Yasuhara, T., & Date, I. (2013). Mannitol enhances therapeutic effects of intra-arterial transplantation of mesenchymal stem cells into the brain after traumatic brain injury. *NEUROSCIENCE LETTERS*, *554*, 156-161.

Papke, R. L., Horenstein, N. A., & Stokes, C. (2015). Nicotinic Activity of Arecoline, the Psychoactive Element of "Betel Nuts", Suggests a Basis for Habitual Use and Anti-Inflammatory Activity. *PLoS One*, *10*(10), e140907.

Park, E., Song, J. H., Kim, M. S., Park, S. H., & Kim, T. S. (2016). Costunolide, a sesquiterpene lactone, inhibits the differentiation of pro-inflammatory CD4(+) T cells through the modulation of mitogen-activated protein kinases. *INTERNATIONAL IMMUNOPHARMACOLOGY*, *40*, 508-516.

Patra, K., Bose, S., Sarkar, S., Rakshit, J., Jana, S., Mukherjee, A., Roy, A., Mandal, D. P., & Bhattacharjee, S. (2012). Amelioration of cyclophosphamide induced myelosuppression and oxidative stress by cinnamic acid. *CHEMICO-BIOLOGICAL INTERACTIONS*, *195*(3), 231-239.

Petrou, S., Mamais, L., Lavranos, G., Tzanetakou, I. P., & Chrysostomou, S. (2018). Effect of Vitamin D Supplementation in Prostate Cancer A Systematic Review of Randomized Control Trials. *Int J Vitam Nutr Res*, *88*(1-2), 100-112.

Pouchieu, C., Chajes, V., Laporte, F., Kesse-Guyot, E., Galan, P., Hercberg, S., Latino-Martel, P., & Touvier, M. (2014). Prospective associations between plasma saturated, monounsaturated and polyunsaturated fatty acids and overall and breast cancer risk - modulation by antioxidants: a nested case-control study. *PLoS One*, *9*(2), e90442.

Prietl, B., Treiber, G., Pieber, T., & Amrein, K. (2013). Vitamin D and Immune Function. *Nutrients*, *5*(7), 2502-2521.

Prorok, T., Jana, M., Patel, D., & Pahan, K. (2019). Cinnamic Acid Protects the Nigrostriatum in a Mouse Model of Parkinson’s Disease via Peroxisome Proliferator-Activated Receptorα. *NEUROCHEMICAL RESEARCH*, *44*(4), 751-762.

Rampersaud, A. M., Dunk, C. E., Lye, S. J., & Renaud, S. J. (2021). Palmitic acid induces inflammation in placental trophoblasts and impairs their migration toward smooth muscle cells through plasminogen activator inhibitor-I. *MOLECULAR HUMAN REPRODUCTION*, *26*(11), 850-865.

Ren, H., He, G., Lu, Z., He, Q., Li, S., Huang, Z., Chen, Z., Cao, C., & Wang, A. (2021). Arecoline induces epithelial-mesenchymal transformation and promotes metastasis of oral cancer by SAA1 expression. *CANCER SCIENCE*, *112*(6), 2173-2184.

Ren, L., Wang, Z., Hua, Q. Z., Xie, H., & Tang, S. Y. (2019). Icaritin prevents vascular calcification in mice. *Journal of Central South University. Medical sciences*, *44*(1), 35-39.

Safholm, J., Manson, M. L., Bood, L., Al-Ameri, M., Orre, A. C., Raud, J., Dahlen, S. E., & Adner, M. (2019). Mannitol triggers mast cell-dependent contractions of human small bronchi and prostacyclin bronchoprotection. *JOURNAL OF ALLERGY AND CLINICAL IMMUNOLOGY*, *144*(4), 984-992.

Saha, I., Das, J., Maiti, B., & Chatterji, U. (2015). A Protective Role of Arecoline Hydrobromide in Experimentally Induced Male Diabetic Rats. *Biomed Research International*, *2015*, 1-12.

Said, S., Neves, F. M., & Griffiths, A. J. F. (2004). Cinnamic acid inhibits the growth of the fungus Neurospora crassa, but is eliminated as acetophenone. *INTERNATIONAL BIODETERIORATION & BIODEGRADATION*, *54*(1), 1-6.

Sarawati, S., Alhaider, A. A., & Abdelgadir, A. M. (2018). Costunolide suppresses an inflammatory angiogenic response in a subcutaneous murine sponge model. *APMIS*, *126*(3), 257-266.

Schmidt, S. D., Costa, A., Rani, B., Nachtigal, E. G., Passani, M. B., Carta, F., Nocentini, A., Myskiw, J. D., Furini, C. R. G., Supuran, C. T., Izquierdo, I., Blandina, P., & Provensi, G. (2020). The role of carbonic anhydrases in extinction of contextual fear memory. *PROCEEDINGS OF THE NATIONAL ACADEMY OF SCIENCES OF THE UNITED STATES OF AMERICA*, *117*(27), 16000-16008.

Serikuly, N., Alpyshov, E. T., Wang, D. W., Wang, J. T., Yang, L. E., Hu, G. J., Yan, D. N., Demin, K. A., Kolesnikova, T. O., Galstyan, D., Amstislavskaya, T. G., Babashev, A. M., Mor, M. S., Efimova, E. V., Gainetdinov, R. R., Strekalova, T., de Abreu, M. S., Song, C., & Kalueff, A. V. (2020). Effects of acute and chronic arecoline in adult zebrafish Anxiolytic-like activity, elevated brain monoamines and the potential role of microglia. *PROGRESS IN NEURO-PSYCHOPHARMACOLOGY & BIOLOGICAL PSYCHIATRY*, *104*.

Shan, R., Zhou, H. Y., Liu, X. F., Su, G. J., Liu, G. S., Zhang, X. L., Sun, C., Yu, Z. N., Zhan, L. F., & Huang, Z. H. (2021). Neuroprotective effects of four different fluids on cerebral ischaemia reperfusion injury in rats through stabilization of the blood-brain barrier. *EUROPEAN JOURNAL OF NEUROSCIENCE*, *54*(4), 5586-5600.

Silwal, P., Lim, K., Heo, J. Y., Park, J. I., Namgung, U., & Park, S. K. (2018). Adenine attenuates lipopolysaccharide-induced inflammatory reactions. *KOREAN JOURNAL OF PHYSIOLOGY & PHARMACOLOGY*, *22*(4), 379-389.

Silwal, P., Shin, K., Choi, S., Kang, S. W., Park, J. B., Lee, H. J., Koo, S. J., Chung, K. H., Namgung, U., Lim, K., Heo, J. Y., Park, J. I., & Park, S. K. (2015). Adenine suppresses IgE-mediated mast cell activation. *MOLECULAR IMMUNOLOGY*, *65*(2), 242-249.

Ślebioda, Z., Szponar, E., & Dorocka-Bobkowska, B. (2016). Vitamin D and Its Relevance in the Etiopathogenesis of Oral Cavity Diseases. *ARCHIVUM IMMUNOLOGIAE ET THERAPIAE EXPERIMENTALIS*, *64*(5), 385-397.

Snigdha, S., Astarita, G., Piomelli, D., & Cotman, C. W. (2012). Effects of diet and behavioral enrichment on free fatty acids in the aged canine brain. *NEUROSCIENCE*, *202*, 326-333.

Su, W. W., Huang, J. Y., Chen, H. M., Lin, J. T., & Kao, S. H. (2020). Adenine inhibits growth of hepatocellular carcinoma cells via AMPK-mediated S phase arrest and apoptotic cascade. *International Journal of Medical Sciences*, *17*(5), 678-684.

Sun, J. (2018). Dietary Vitamin D, Vitamin D Receptor, and Microbiome. *HHS Public Access*, *21*(6), 471-474.

Suzuki, S., Qiu, H. L., Okada, A., Kasama, T., Ohta, K., Warita, K., Tanaka, K., Miki, T., & Takeuchi, Y. (2012). BDNF-Dependent Accumulation of Palmitoleic Acid in CNS Neurons. *CELLULAR AND MOLECULAR NEUROBIOLOGY* (32), 1367-1373.

Szkudelski, T., & Szkudelska, K. (2015). Regulatory role of adenosine in insulin secretion from pancreatic beta-cells-Action via adenosine A (1) receptor and beyond. *JOURNAL OF PHYSIOLOGY AND BIOCHEMISTRY*, *71*(1), 133-140.

Tajiri, N., Lee, J. Y., Acosta, S., Sanberg, P. R., & Borlongan, C. V. (2016). Breaking the Blood-Brain Barrier With Mannitol to Aid Stem Cell Therapeutics in the Chronic Stroke Brain. *CELL TRANSPLANTATION*, *25*(8), 1453-1460.

Talbot, N. A., Wheeler-Jones, C. P., & Cleasby, M. E. (2014). Palmitoleic acid prevents palmitic acid-induced macrophage activation and consequent p38 MAPK-mediated-skeletal muscle insulin resistance. *MOLECULAR AND CELLULAR ENDOCRINOLOGY*, *393*(1-2), 129-142.

Tang, Y. H., Jiang, Y., Meng, J. S., & Tao, J. (2018). A brief review of physiological roles, plant resources, synthesis, purification and oxidative stability of Alpha-linolenic Acid. *Emirates Journal of Food and Agriculture*, *30*(5), 341-356.

Tello Ortiz, A., & Contreras Taboada, A. (1959). Adenine in therapy of agranulocytosis. *La adenina en el tratamiento de la agranulocitosis.*, *21*(6), 305-309.

Terai, K., Mizukami, K., & Okada, M. (2008). Comparison of chronic renal failure rats and modification of the preparation protocol as a hyperphosphataemia model. *NEPHROLOGY*, *13*(2), 139-146.

Thongrong, C., Kong, N., Govindarajan, B., Allen, D., Mendel, E., & Bergese, S. D. (2014). Current Purpose and Practice of Hypertonic Saline in Neurosurgery: A Review of the Literature. *World Neurosurgery*, *82*(6).

Tian, H. W., Li, Y. P., & Zhang, J. (2021). Dioscorea deltoidei (Dioscoreaceae) leaf extract exerts anti-atherosclerotic effect in rats via down-regulation of phosphorylated JAK STAT. *TROPICAL JOURNAL OF PHARMACEUTICAL RESEARCH*, *20*(9), 1941-1947.

Titov, V. N., Krylin, V. V., & Shiriaeva, I. K. (2011). Prevention of atherosclerosis. Excess of palmitic acid in food--a cause of hypercholesterolemia, inflammatory syndrome, insulin resistance in myocytes, and apoptosis. *Klinicheskaia laboratornaia diagnostika* (2), 4-15.

Tsai, Y. W., Lu, C. H., Chang, R. C. A., Hsu, Y. P., Ho, L. T., & Shih, K. C. (2021). Palmitoleic acid ameliorates palmitic acid-induced proinflammation in J774A.1 macrophages via TLR4-dependent and TNF-alpha-independent signallings. *PROSTAGLANDINS LEUKOTRIENES AND ESSENTIAL FATTY ACIDS*, *169*.

Tu, T. H., Kim, H., Yang, S., Kim, J. K., & Kim, J. G. (2019). Linoleic acid rescues microglia inflammation triggered by saturated fatty acid. *BIOCHEMICAL AND BIOPHYSICAL RESEARCH COMMUNICATIONS*, *513*(1), 201-206.

Turina, M., Mulhall, A., Gardner, S., C., P. J. H., & N., M. F. (2008). Mannitol Upregulates Monocyte HLA-DR, Monocyte and Neutrophil CD11b, and Inhibits Neutrophil Apoptosis. *INFLAMMATION*, *31*(2), 74-83.

Vesga-Jimenez, D. J., Martin, C., Barreto, G. E., Aristizabal-Pachon, A. F., Pinzon, A., & Gonzalez, J. (2022). Fatty Acids an Insight into the Pathogenesis of Neurodegenerative Diseases and Therapeutic Potential. *INTERNATIONAL JOURNAL OF MOLECULAR SCIENCES*, *23*(5).

Waki, M., Ide, Y., Ishizaki, I., Nagata, Y., Masaki, N., Sugiyama, E., Kurabe, N., Nicolaescu, D., Yamazaki, F., Hayasaka, T., Ikegami, K., Kondo, T., Shibata, K., Hiraide, T., Taki, Y., Ogura, H., Shiiya, N., Sanada, N., & Setou, M. (2014). Single-cell time-of-flight secondary ion mass spectrometry reveals that human breast cancer stem cells have significantly lower content of palmitoleic acid compared to their counterpart non-stem cancer cells. *BIOCHIMIE*, *107*, 73-77.

Wang, H., Cao, F. L., Li, J. M., Li, Y., Shi, C., Lan, W. J., Li, D. D., Zhao, H., Zhang, Y., Zhang, Z., Liu, X. H., Meng, R., Yang, B. F., & Zhou, J. (2014). Arsenic trioxide and mannitol for the treatment of acute promyelocytic leukemia relapse in the central nervous system. *BLOOD*, *124*(12), 1998-2000.

Wang, L. L., Liu, X., Hou, J., Wei, D. D., Liu, P. L., Fan, K. L., Zhang, L., Nie, L. T., Li, X., Huo, W. Q., Jing, T., Li, W. J., Wang, C. J., & Mao, Z. X. (2021). Serum Vitamin D Affected Type 2 Diabetes though Altering Lipid Profile and Modified the Effects of Testosterone on Diabetes Status. *Nutrients*, *13*(1).

Wang, L., Gu, L., & Tang, Z. (2019). Cytokines secreted by arecoline activate fibroblasts that affect the balance of TH17 and Treg. *JOURNAL OF ORAL PATHOLOGY & MEDICINE*, *49*(2), 156-163.

Watanabe, S., Yoshimi, Y., & Ikekita, M. (2003). Neuroprotective effect of adenine on purkinje cell survival in rat cerebellar primary cultures. *JOURNAL OF NEUROSCIENCE RESEARCH*, *74*(5), 754-759.

Weltha, L., Reemmer, J., & Boison, D. (2019). The role of adenosine in epilepsy. *BRAIN RESEARCH BULLETIN*, *151*, 46-54.

Wu, T. T., Chen, C. C., Lin, J. T., Young, G. H., Wang, H. C., & Chen, H. M. (2019). The anti-inflammatory function of adenine occurs through AMPK activation and its downstream transcriptional regulation in THP-1 cells. *BIOSCIENCE BIOTECHNOLOGY AND BIOCHEMISTRY*, *83*(12), 2220-2229.

Yanguas-Casas, N., Crespo-Castrillo, A., De, C. M. L., Chowen, J. A., Azcoitia, I., Arevalo, M. A., & Garcia-Segura, L. M. (2018). Sex differences in the phagocytic and migratory activity of microglia and their impairment by palmitic acid. *GLIA*, *66*(3), 522-537.

Yoon, W. J., Kim, M. J., Moon, J. Y., Kang, H. J., Kim, G. O., Lee, N. H., & Hyun, C. G. (2010). Effect of palmitoleic acid on melanogenic protein expression in murine b16 melanoma. *Journal of Oleo Science*, *59*(6), 315-319.

Young, G. H., Lin, J. T., Cheng, Y. F., Huang, C. F., Chao, C. Y., Nong, J. Y., Chen, P. K., & Chen, H. M. (2015). Identification of adenine modulating AMPK activation in NIH 3T3 cells by proteomic approach. *Journal of Proteomics*, *120*, 204-214.

Yu, Y. P., Li, C., Liu, J. X., Zhu, F. Y., Wei, S. N., Huang, Y. H., Huang, X. H., & Qin, Q. W. (2020). Palmitic Acid Promotes Virus Replication in Fish Cell by Modulating Autophagy Flux and TBK1-IRF3/7 Pathway. *Frontiers in Immunology*, *11*.

Yuan, S., & Larsson, S. C. (2022). Inverse Association Between Serum 25-Hydroxyvitamin D and Nonalcoholic Fatty Liver Disease. *Clinical Gastroenterology and Hepatology*.

Zelzer, S., Hofer, E., Meinitzer, A., Fritz-Petrin, E., Simstich, S., Goessler, W., Schmidt, R., & Herrmann, M. (2021). Association of vitamin D metabolites with cognitive function and brain atrophy in elderly individuals-the Austrian stroke prevention study. *Aging-US*, *13*(7), 9455-9467.

Zhang, W. Y., Franco, D. A., Schwartz, E., D'Souza, K., Karnick, S., & Reaven, P. D. (2017). HDL inhibits saturated fatty acid mediated augmentation of innate immune responses in endothelial cells by a novel pathway. *ATHEROSCLEROSIS*, *259*, 83-96.

Zhang, Y. Q., Wang, X. D., Wu, Q., Wang, H., Zhao, L., Wang, X. H., Mu, M. D., Xie, E. J., He, X. Y., Shao, D. D., Shang, Y. N., Lai, Y. R., Ginzburg, Y., Min, J. X., & Wang, F. D. (2018). Adenine alleviates iron overload by cAMP PKA mediated hepatic hepcidin in mice. *JOURNAL OF CELLULAR PHYSIOLOGY*, *233*(9), 7268-7278.

Zong, G., Ye, X., Sun, L., Li, H., Yu, Z., Hu, F. B., Sun, Q., & Lin, X. (2012). Associations of erythrocyte palmitoleic acid with adipokines, inflammatory markers, and the metabolic syndrome in middle-aged and older Chinese. *The American Journal of Clinical Nutrition*, *96*(5), 970-976.

Zorgetto-Pinheiro, V. A., Machate, D. J., Figueiredo, P. S., Marcelino, G., Hiane, P. A., Pott, A., Guimaraes, R. D. A., & Bogo, D. (2022). Omega-3 Fatty Acids and Balanced Gut Microbiota on Chronic Inflammatory Diseases a Close Look at Ulcerative Colitis and Rheumatoid Arthritis Pathogenesis. *JOURNAL OF MEDICINAL FOOD*, *25*(4), 341-354.

Zuo, B., Zhu, J., Xiao, F., Wang, C., Shen, Y., & Chen, X. (2020). Identification of novel biomarkers and candidate small molecule drugs in rheumatoid arthritis and osteoarthritis based on bioinformatics analysis of high-throughput data. *BIOSCIENCE REPORTS*, *40*(12).
